# A chimeric antigen receptor tailored to integrate complementary activation signals potentiates the antitumor activity of NK cells

**DOI:** 10.1186/s13046-025-03351-5

**Published:** 2025-03-06

**Authors:** Eunbi Yi, Eunbi Lee, Hyo Jin Park, Hyeon Ho Lee, So Hyeon Yun, Hun Sik Kim

**Affiliations:** 1 Department of Microbiology, Brain Korea 21 Project, Asan Medical Center, University of Ulsan College of Medicine, Seoul, South Korea; 2 Stem Cell Immunomodulation Research Center, University of Ulsan College of Medicine, Seoul, Republic of Korea

**Keywords:** Natural killer cells, Chimeric antigen receptor, Synergistic signaling, DAP10, 2B4

## Abstract

**Background:**

Chimeric antigen receptors (CARs) are synthetic receptors that reprogram the target specificity and functions of CAR-expressing effector cells. The design of CAR constructs typically includes an extracellular antigen-binding moiety, hinge (H), transmembrane (TM), and intracellular signaling domains. Conventional CAR constructs are primarily designed for T cells but have been directly adopted for other effector cells, including natural killer (NK) cells, without tailored optimization. Given the benefits of CAR-NK cells over CAR-T cells in terms of safety, off-the-shelf utility, and antigen escape, there is an increasing emphasis on tailoring them to NK cell activation mechanisms.

**Methods:**

We first have taken a stepwise approach to modifying CAR components such as the combination and order of the H, TM, and signaling domains to achieve such tailoring in NK cells. Functionality of NK-tailored CARs were evaluated in vitro and in vivo in a model of CD19-expressing lymphoma, along with their expression and signaling properties in NK cells.

**Results:**

We found that NK-CAR driven by the synergistic combination of NK receptors NKG2D and 2B4 rather than DNAM-1 and 2B4 induces potent activation in NK cells. Further, more effective CAR-mediated cytotoxicity was observed following the sequential combination of DAP10, but not NKG2D TM, with 2B4 signaling domain despite the capacity of NKG2D TM to recruit endogenous DAP10 for signaling. Accordingly, an NK-CAR incorporating DAP10, 2B4, and CD3ζ signaling domains coupled to CD8α H and CD28 TM domains was identified as the most promising candidate to improve CAR-mediated cytotoxicity. This NK-tailored CAR provided more potent antitumor activity than a conventional T-CAR when delivered to NK cells both in vitro and in vivo.

**Conclusions:**

Hence, NK receptor-based domains hold great promise for the future of NK-CAR design with potentially significant therapeutic benefits.

**Supplementary Information:**

The online version contains supplementary material available at 10.1186/s13046-025-03351-5.

## Background

Chimeric antigen receptors (CARs) are synthetic receptors comprising an extracellular (EC) antigen binding and hinge (H) domain, transmembrane (TM) domain, and intracellular signaling domains that were originally developed to redirect the specificity of T cells [1, 2]. The first generation CARs contained a tumor associated antigen targeting single-chain variable fragment (scFv) coupled to signaling CD3ζ or FcεRIγ chain and could activate T cells without major histocompatibility complex (MHC) restriction but did not enable T cell proliferation or survival in clinical trials [3]. To improve their cytotoxicity and persistence, next generation CARs incorporated one (second generation) or two (third generation) costimulatory domains between the TM and signaling CD3ζ chain [4–6]. The commonly used costimulatory domains are CD28 and/or 4-1BB (CD137), and the second generation CARs targeting CD19 showed remarkable and durable efficacy in patients with lymphoma and leukemia of B cell origin [7, 8].

Although such favorable outcomes have prompted the growth of CAR-T clinical trials, there are notable limitations of these agents, including CAR-T cell-related toxicities (e.g., cytokine release syndrome and neurotoxicity), off-the-shelf utility, and target antigen escape [9–12]. In addition, CAR-T cell therapy with autologous patient T cells requires high cost and length of manufacturing but is often challenged with a significant manufacturing failure rate depending on patient’s clinical condition [13–15]. Accordingly, allogeneic CAR-T cells from healthy donors have been developed but this requires an additional gene-editing process to eliminate the signaling or expression of donor T-cell receptor (TCR) for preventing severe side effects such as graft-versus-host disease (GvHD) [14]. However, the generation of universal CAR-T cells have potential drawbacks related to chromosomal abnormalities and manufacturing complexities that impact cell fitness and/or yield [16].

To overcome these limitations of CAR-T cells, there is a growing interest in exploring natural killer (NK) cells as an attractive CAR driver candidate for off-the-shelf immunotherapy of cancer [17–19]. NK cells are key innate effectors in cancer immunosurveillance and are effective in eliminating metastatic cancer and cancer stem cells in an antigen-independent manner [20–22]. Notably, the significant therapeutic benefits without the risk of GvHD of allogeneic donor NK cells have been demonstrated [23–25]. Accordingly, off-the-shelf NK and CAR-NK strategies can be developed from diverse cellular sources, including peripheral and cord blood from healthy donors, induced pluripotent stem cells (iPSCs), and NK cell lines [24, 25]. Moreover, the downregulation of CAR target antigen by cancer cells can be overcome by the intrinsic cytotoxic capacity of NK cells. However, CAR constructs currently used to engineer NK cells are primarily designed for T cells and are not optimized for NK cell activation signals [18, 26, 27]. Improving the therapeutic efficacy of CAR-NK cells will therefore require the structural design of CAR molecules to be tailored to NK cell activation mechanisms.

Distinct from T cell activation that are dominated by an MHC-restricted TCR, NK cell activation relies on an array of diverse activating receptors with distinct cytoplasmic signaling motifs including NKG2D (CD314), 2B4 (CD244), DNAM-1 (CD226), CD16, and natural cytotoxicity receptors (NCRs) such as NKp30, NKp44, and NKp46 [28–30]. Apart from CD16, no single activating receptor is sufficient to activate natural cytotoxicity but requires complementary combination of other activating receptors such as co-engagement of 2B4 with NKG2D or 2B4 with DNAM-1 but not NKG2D with DNAM-1 [29, 31, 32]. These receptor combinations trigger cytotoxic responses of NK cells through immunoreceptor tyrosine-based activation motif (ITAM; consensus sequence YxxL/I, where x represents any amino acid)-independent pathways unlike ITAM-coupled receptors (e.g., NCRs and CD16) [29]. The ITAM-bearing CD3ζ and FcεRIγ chains associate with NKp46 and CD16 by forming either homodimers or heterodimers and recruit Syk family kinases for activation upon tyrosine phosphorylation of the ITAM. NKp46 can cooperate with NKG2D, 2B4, or DNAM-1 for the synergistic activation of NK cells but does not efficiently enhance CD16-mediated activation [31]. NKG2D associates with the TM adaptor protein DAP10 that harbors an immunoglobulin tyrosine tail (ITT) motif (consensus sequence YxxM) like CD28 [33, 34], whereas 2B4 contains cytoplasmic immunoreceptor tyrosine-based switch motif (ITSM; consensus sequence TxYxxV/I) and signals through the activating adaptor SAP upon tyrosine phosphorylation of the ITSM [35]. Similar to NKG2D, DNAM-1 promotes activation via an ITT-like motif [36].

Given the unique pattern of synergistic receptors that use different signaling modules, we speculate that an appropriate combination of distinct signaling modules is required for the optimal activation of NK cells and could be adapted for the design of NK-tailored CAR. In general, the CD3ζ chain has been used as the main signaling domain and coupled to the cytoplasmic signaling domain and/or adaptor protein from NK receptors including DAP10, the cytoplasmic domain of 2B4 and DNAM-1 [18, 37–40]. In addition, the TM domains of NKG2D and NCRs that can associate with their cognate adaptor proteins have been previously incorporated in the screening of CAR constructs [26, 41]. However, the benefits of incorporating NK receptor-based domains over conventional domains in CAR construction have not been systematically studied but evaluated thus far using a limited set of domain combinations [18, 37–40]. Accordingly, the optimal combination of activation domains, along with their sequential order, for the design of NK-tailored CAR has remained to be determined.

In this study, we sought to assess the impacts of systematic combinations of the H, TM domain, and signaling domain on the function of CAR-bearing NK cells, with a focus on NK-specific receptor synergy. The results revealed that a CAR construct combining the signaling domains of DAP10, 2B4, and CD3ζ as well as the CD8α H and CD28 TM domain was the most promising candidate to improve CAR-NK cell-mediated cytotoxicity. Compared to conventional T-CAR that is driven by CD28 and CD3ζ signaling domains, this NK-tailored CAR produced a more potent antitumor cytotoxic activity in NK cells, both in vitro and in vivo, in a model of CD19-expressing lymphoma. Moreover, the sequential combination of DAP10 and 2B4 signaling domains (DAP10-2B4) was more effective than that of NKG2D TM and 2B4 signaling domain (NKG2D TM-2B4), or that of 2B4 and DAP10 signaling domains (2B4-DAP10), in promoting the synergistic activation of NK cells, highlighting the importance of proper combination and order of activation domains. Thus, our present findings support the premise that NK receptor-based domains have utility for improving NK-CAR designs and provide significant insights into the rational design of optimized NK-CARs.

## Methods

### Cells and culture

The human NK cell line NKL (gift of M. Robertson, Indiana University Medical Center, Indianapolis, IN) was maintained in Roswell Park Memorial Institute-1640 (RPMI-1640) (Gibco) medium containing 10% fetal bovine serum (FBS) (Gibco), 1% penicillin/streptomycin (Gibco), 1 mM sodium pyruvate (Gibco) and 200 U/mL recombinant IL-2 (rIL-2) (Roche). NKL cells were rested to reproduce receptor synergy in RPMI-1640 containing 5% FBS, 0.5% penicillin/streptomycin, 0.5 mM sodium pyruvate without rIL-2 for 24 h [32]. The human NK cell line NK92 (American Type Culture Collection) was maintained in Minimum Essential Medium-α (MEM-α) (Gibco) containing 20% FBS, 1% penicillin/streptomycin, 1% vitamin solution (Gibco), 0.1 mM β-mercaptoethanol (Gibco) and 200 U/ml rIL-2. REH [Acute Lymphocytic Leukemia (ALL), CD19^+^, CD20^−^] (American Type Culture Collection), Raji [Burkitt’s Lymphoma, CD19^+^, CD20^+^] (American Type Culture Collection) and Ramos [Burkitt’s Lymphoma, CD19^+^, CD20^+^] (American Type Culture Collection) cells were maintained in RPMI-1640 containing 10% FBS, 1% penicillin/streptomycin. The K562 cell line (American Type Culture Collection) was maintained in Iscove's Modified Dulbecco's Medium (IMDM) (HyClone) containing 10% FBS, 1% penicillin/streptomycin. A platinum-A retroviral packaging cell line (Plat-A; Cell Biolabs) were maintained in Dulbecco's Modified Eagle Medium (DMEM) (Gibco) containing 10% FBS, 1% penicillin/streptomycin, 1 μg/mL puromycin (Sigma) and 10 μg/mL blasticidin (Gibco). All cells were cultured in a 5% CO_2_ incubator at 37 °C and were confirmed to be mycoplasma-free by using mycoplasma PCR detection kit (MP Biomedicals).

### CAR constructs

All anti-CD19 CAR constructs harbor the CD8α signal peptide (SP) for CAR secretion, Myc tag (EQKLISEEDL) for analysis of cell surface expression, and mouse anti-human CD19 single-chain variable fragment (scFv) for antigen binding in common, and also contain a hinge (H) and/or extracellular (EC) domain, transmembrane (TM) domain both of which link antigen recognition domain to intracellular signaling domain, and cytoplasmic signaling domains (CYP), as indicated in Table 1. Each CAR is designed with a combination of a H and/or EC of CD8α (NM_001145873.1, a.a.: 138–182), CD28 (NM_006139.4, a.a.: 114–152), DAP10 (NM_014266.4, a.a.: 19–48) and/or NKG2D (NM_007360.4, a.a.:90–80), TM of CD28 (NM_006139.4, a.a.: 153–179), DAP10 (NM_014266.4, a.a.: 49–69), NKG2D (NM_007360.4, a.a.: 79–48), 2B4 (NM_016382.4, a.a.: 222–245) or DNAM-1 (NM_001303619.2, a.a.: 93–120), and CYP of CD28 (NM_006139.4, a.a.: 180–220), DAP10 (NM_014266.4, a.a.: 70–93), NKG2D (NM_007360.4, a.a.: 47–1), 2B4 (NM_016382.4, a.a.: 246–365), DNAM-1 (NM_001303619.2, a.a.: 121–181) and/or CD3ζ (NM_000734.3, a.a.:52–163), as indicated with amino acid sequences used for CAR domains in Supplementary Table 1. The full-length NK-CAR constructs were generated through the fusion of two PCR fragments corresponding to anti-CD19 scFv and signaling moieties, respectively, by overlap extension PCR (OE-PCR) (Supplementary Fig. 1). The CD8α SP, Myc tag, and anti-CD19 scFv sequences were obtained from pHR_PGK_anti-CD19_SynNotch_Gal4VP64 (Addgene #79,125). A PCR fragment encoding these sequences was then amplified using the primers with a 20 bp overlapping DNA sequence with the CD8α SP and anti-CD19 scFv, respectively. Another PCR fragment encoding the H and/or EC, TM, and CYP domains was amplified from the plasmid synthesized for NK-CARs using the primers with a 20 bp overlapping DNA sequence with the H and CYP, respectively. These two separate PCR fragments were combined and then connected using OE-PCR with final primers containing EcoR1 and Xho1 restriction site, respectively, for cloning of CAR construct. The sequence encoding the full-length NK-CARs was cloned into the EcoR1 and Xho1 sites of the pMXs-IRES (internal ribosome entry site)-EGFP (enhanced green fluorescent protein) retroviral vector (Cell Biolabs) that allows the co-expression of CAR and EGFP under the control of the same promoter for the detection and sorting of CAR-expressing NK cells. In detail, the sequences of NK-CAR1, NK-CAR2, NK-CAR2-1, NK-CAR3, NK-CAR4, NK-CAR5, NK-CAR7-1, and NK-CAR10-1 were synthesized using GenScript or IDT. Using PCR, NK-CAR6 was obtained by replacing CD8α (H) of NK-CAR1 with CD28 (H). NK-CAR7 and NK-CAR8 were obtained by replacing CD8α (H) of NK-CAR2 with CD28 (H) or NKG2D (H), respectively. The NK-CAR6-1 and NK-CAR6-2 constructs were obtained from NK-CAR6 via the deletion of CD28 (H) and DAP10 (EC), respectively. The NK-CAR6-3 construct was obtained from NK-CAR1 by the deletion of DAP10 (EC). NK-CAR9 and NK-CAR10 were obtained from NK-CAR1 via the deletion of DAP10 (EC) and the replacement of DAP10 (TM) with CD28 (TM) in common and additional change of CD8α (H) to CD28 (H) for NK-CAR9. NK-CAR11 and NK-CAR12 were obtained from NK-CAR6 and NK-CAR10 via the addition of CD3ζ (CYP). All CAR constructs were confirmed by sequencing analyses (Cosmogenetech). The empty vector pMXs-IRES-EGFP was used as a control (EV).

**Table 1:** List of CAR constructs used in this study

CAR	Target	Construct structure (H and/or EC-TM-CYP domain)	Index (First shown)
T-CAR1	CD19	CD8α(H)-CD28(TM)-CD3ζ	Figure 1
T-CAR2	CD19	CD28(H)-CD28(TM)-CD3ζ	Figure 2
T-CAR3	CD19	CD28(H)-CD28(TM)-CD28-CD3ζ	Figure 4
NK-CAR1	CD19	CD8α(H)-DAP10(EC)-DAP10(TM)-DAP10-2B4	Figure 1
NK-CAR2	CD19	CD8α(H)-NKG2D(TM)−2B4	Figure 1
NK-CAR2-1	CD19	CD8α(H)-NKG2D(TM)-NKG2D-2B4	Figure S2
NK-CAR3	CD19	CD8α(H)−2B4(TM)−2B4-DNAM1	Figure 1
NK-CAR4	CD19	CD8α(H)-DNAM1(TM)-DNAM1-2B4	Figure 1
NK-CAR5	CD19	CD8α(H)-CD28(TM)-CD28-2B4	Figure 1
NK-CAR6	CD19	CD28(H)-DAP10(EC)-DAP10(TM)-DAP10-2B4	Figure 2
NK-CAR6-1	CD19	DAP10(EC)-DAP10(TM)-DAP10-2B4	Figure 3
NK-CAR6-2	CD19	CD28(H)-DAP10(TM)-DAP10-2B4	Figure 3
NK-CAR6-3	CD19	CD8α(H)-DAP10(TM)-DAP10-2B4	Figure 3
NK-CAR7	CD19	CD28(H)-NKG2D(TM)−2B4	Figure 2
NK-CAR7-1	CD19	CD28(H)-NKG2D(TM)-NKG2D-2B4	Figure S2
NK-CAR8	CD19	NKG2D(H)-NKG2D(TM)−2B4	Figure 2
NK-CAR9	CD19	CD28(H)-CD28(TM)-DAP10-2B4	Figure 4
NK-CAR10	CD19	CD8α(H)-CD28(TM)-DAP10-2B4	Figure 4
NK-CAR10-1	CD19	CD8α(H)-CD28(TM)- 2B4-DAP10	Figure S3
NK-CAR11	CD19	CD28(H)-DAP10(EC)-DAP10(TM)-DAP10-2B4-CD3ζ	Figure 5
NK-CAR12	CD19	CD8α(H)-CD28(TM)-DAP10-2B4-CD3ζ	Figure 5

*Abbreviations*: *CAR* Chimeric antigen receptor, *H* Hinge, *EC* Extracellular domain, *TM* Transmembrane domain, *CYP* Cytoplasmic signaling domain

### CAR-NK cell production

To produce retroviruses, 0.5 × 10^6^ Plat-A cells were cultured for 16 h and then transfected with 2 μg of EV or pMXs-CAR-IRES-EGFP retroviral vector using X-tremeGENE9 (Roche). At 24 h post-transfection, the medium was replaced with fresh Plat-A cell culture medium without selection markers. The following day, retroviral supernatants were harvested and mixed with fresh NK cell line culture medium at a 1:1 ratio. For transduction of human NK cell lines, aliquots of 0.5 × 10^6^ human NK cell line (NKL or NK92) were then resuspended with 2.4 mL of virus-containing medium in the presence of 10 μg/mL hexadimethrine bromide (Sigma) and 200 U/mL rIL-2, transferred to a 12-well plate, and centrifuged (700 × g, 32℃, 30 min). After 3 h incubation in a CO_2_ incubator at 37℃, the NK cell lines were centrifuged (700 × g, 32℃, 30 min). After a further 3 h incubation, the viral supernatants were removed and replaced with fresh culture medium containing 200 U/mL rIL-2. Following transduction with differing efficiencies (~ 24% to ~ 43%), GFP-expressing NK cells were sorted using a FACSAria III cell sorter (BD Biosciences). For transduction of primary human NK cells, NK cells in peripheral mononuclear cells obtained from healthy volunteers were first expanded upon stimulation with K562-mb15-41BBL cell line (gift of D. Campana, National University of Singapore) in Stem Cell Growth Medium (SCGM; CellGenix) supplemented with 10% FBS and 10 U/mL rIL-2 as previously described [42, 43]. On day 7 of NK cell expansion, aliquots of 1 × 10^6^ human NK cells were resuspended with 2.4 mL of retroviral supernatant and 2.4 mL of fresh SCGM (10% FBS) in the presence of 20 μg/mL RetroNectin (Takara), 100 U/mL rIL-2, and 5 ng/mL rIL-15 (PeproTech), transferred to a 6-well plate (Nunc), and centrifuged (1,200 × g, 32℃, 90 min). After 24 h incubation in a CO_2_ incubator at 37℃, the viral supernatants were removed and replaced with fresh SCGM containing 10% FBS, 100 U/mL rIL-2, and 5 ng/mL rIL-15 for an additional week with a medium exchange being made every 2 days for functional study thereafter.

### CD19 knock-out (KO) REH cell production

To generate CD19 KO cells using CRISPR–Cas9 gene editing, two different short guide RNAs (sgRNAs) against CD19 were synthesized. The sgRNA target CD19 sequences are 5′-AAGCGGGGACTCCCGAGACC-3′ for sgRNA #1 and 5′-GGTTCAGGCTGTCCCTCGGT-3′ for sgRNA #2. sgRNAs were synthesized with 2′-O-methyl 3′ phosphorothioate modifications in the first and last three nucleotides (Synthego). To generate CD19 KO cells, REH cells (1.2 × 10^6^) were resuspended in Amaxa solution V (Lonza, 100 μL) with a mixture of ribonucleoprotein (RNP) including the final 100 μM of sgRNA, 61 μM of Cas9 (IDT, 1081061) and 100 μM of electroporation Enhancer to improve the rate of RNP delivery into cells (IDT, 1075915) and then transfected using the program X-001 Amaxa Nucleofector II system. The cells were then seeded, incubated in the culture media for 24 h, and thereafter subject to media exchange for 48 h. After these incubations, the success of the CD19 KO in REH cells was assessed by surface CD19 expression using flow cytometry.

### Flow cytometry

To measure CAR surface expression, CAR-NK cells (0.2 × 10^6^ cells / 100 μL) were stained with anti-Myc tag-Alexa Fluor 647 (clone 9B11, Cell Signaling) for 1 h in the dark at 4 °C. The CD19 surface expression levels in the target cells (0.2 × 10^6^ cells / 100 μL) were determined by staining with anti-CD19-phycoerythrin (PE) (clone 4G7, BD Bioscience) for 1 h in the dark at 4 °C. After staining, the cells were washed twice for 3 min at room temperature with FACS buffer (1 × DPBS with 1% FBS) and analyzed using flow cytometry. To measure CAR intracellular accumulation, CAR-NK cells were fixed and permeabilized with BD Cytofix/Cytoperm solution (BD Bioscience) for 20 min in the dark at 4 °C. The cells were then washed twice with BD Perm/Wash buffer solution (BD Bioscience) and stained with anti-Myc tag-Alexa Fluor 647 for 1 h in the dark at 4 °C (0.2 × 10^6^ cells / 100 μL). After staining, the cells were washed twice for 3 min at room temperature with BD Perm/Wash buffer solution and analyzed using flow cytometry. All samples were acquired on a BD Accuri C6 (BD Biosciences) and analyzed with FlowJo software (Tree Star). As a control, unstained CAR-NK cells as well as stained parent and EV-NK cells were analyzed with the same procedure. CAR expression was assessed by means of the mean fluorescence intensity (MFI) of CAR-NK cells relative to EV-NK cells following the sorting of GFP-expressing NK cells.

### Confocal microscopy analysis of CAR expression via Myc tag detection

EV-NKL or CAR-NKL cells were plated onto poly-L-lysine (Sigma, #4832)-coated sterile glass cover slips in 12-well plates (Nunc). These NKL cells (0.25 × 10^6^) were attached for 30 min at 37 °C in complete RPMI-1640 medium without serum and then fixed with 4% (w/v) paraformaldehyde (PFA) in DPBS (Gibco) for 30 min at room temperature and washed with DPBS. Additionally, to confirm the intracellular accumulation of CAR, the cells were permeabilized with DPBS containing 1% (w/v) BSA (Sigma), 0.2% (v/v) Triton X-100 (Sigma), and 0.1% (w/v) sodium citrate (Sigma) for 10 min at room temperature and washed with DPBS. The cells were then blocked with DPBS containing 1% (w/v) BSA and 1% (v/v) goat serum (Invitrogen, #31872) for 30 min at room temperature and washed with DPBS. This was followed by incubation with mouse anti-Myc tag antibody (Thermo, clone 9E10; 1 µg/mL in blocking solution; overnight at 4 °C), washing three times with DPBS, and staining with goat anti-mouse-Alexa Fluor 647 (Jackson Immunoresearch; 1:100 in blocking solution; 90 min at room temperature) and DAPI (1:2000 in blocking solution; 2 min at room temperature) in the dark. The cells were finally washed three times with DPBS before being mounted onto slides using ProLong Gold antifade reagent (Invitrogen, #P36930) covered with aluminium foil. Samples were dried overnight in the dark and imaged under a LSM880 confocal fluorescence microscope (Zeiss) using a × 63 oil immersion objective. The following laser lines and filters were used: 405 nm (30 mW) laser line and 445/35 nm filter for DAPI detection; 633 nm (5 mW) laser line and 665/30 nm filter for Alexa Fluor 647 detection.

### Cell lysis assay

To assess the cytotoxicity of CAR-NKL without pre-stimulation of cytokines, CAR-NKL cells after 48 h of post-culture were washed with serum-free RPMI and rested in RPMI containing 5% FBS, 0.5% penicillin/streptomycin, 0.5 mM sodium pyruvate without rIL-2 for 24 h at 37 °C. CAR-NK92 cells were used in the experiment after 48 h of post-culture without IL-2 starvation. Briefly, target cells were separately loaded with 40 μM DELFIA BATDA (Revvity) for 30 min at 37℃, washed twice with medium containing 1 mM sulfinpyrazone (Sigma), and then co-incubated at 37 °C in triplicate at 5,000 cells/well with effector cells at the indicated effector to target (E:T) ratios for 2 h (CAR-NKL) or 30 min (CAR-NK92). Total lysis control was achieved with 2% Triton X-100. After a period of incubation, the plates were centrifuged at 1,500 rpm for 3 min, and the supernatants were added to a 20% europium solution (Revvity) containing 0.3 M acetic acid (Sigma) and incubated for 5 min. Fluorescence was detected using Victor X4 multi-label plate reader (PerkinElmer) by means of the standard europium TRF protocol (340 nm excitation and 616/9 nm emission). The specific lysis percentage (%) was determined by: (test release – spontaneous release) / (maximum release—spontaneous release) × 100.

### Bead stimulation and cell mixing

To determine the activation of Akt and Erk1/2 in CAR-NKL cells, recombinant human (rh) CD19 Fc chimera (R&D, #9269-CD) and REH cells were used as stimulators. For rhCD19 Fc chimera stimulation, protein G dynabeads (4 × 10^7^; Invitrogen, #1003D) were coated with the rhCD19 Fc chimera (4 μg) in PBS containing 1% FBS and 0.01% Tween-20 for 1 h at 4 °C and suspended in RPMI containing 5% FBS to prepare a bead solution. The bead solution (4 × 10^7^) and NKL cells (5 × 10^6^) were mixed, incubated for 10 min on ice and then incubated at 37 °C for 2 min or 5 min. For cell mixing experiments, REH cells (5 × 10^6^) and NKL cells (5 × 10^6^) were separately chilled on ice for 10 min and then mixed. The cells were then further incubated for 10 min on ice and then incubated at 37 °C for 2 min or 5 min. The cells were again placed in ice and lysed using lysis buffer [50 mM Tris–HCl (pH 7.5), 150 mM NaCl, 1% Triton X-100, 5 mM EDTA, 1 mM NaVO_3_, 50 mM NaF, 1 mM phenylmethylsulfonyl fluoride (PMSF) and protease inhibitor cocktail (Thermo)] for 30 min. Cell debris was removed by centrifugation, and the supernatants were recovered for further analysis. Lysates were resuspended in 1 × NuPAGE LDS sample buffer (Invitrogen) containing 50 mM dithiothreitol (Sigma), and further incubated for 10 min at 70 °C for immunoblotting analysis.

### Immunoprecipitation

For detection of the interaction of NK-CARs with endogenous NKG2D, NKL or CAR-NKL cell lysates were prepared with a NP40 lysis buffer [50 mM Tris–HCl (pH 7.5), 150 mM NaCl, 1% NP-40, 5 mM EDTA, 1 mM NaVO3, 50 mM NaF, 1 mM PMSF, and protease inhibitor cocktail (Thermo)] as described [32]. Cell lysates were precleared with protein G dynabeads (0.75 mg, Thermo) for 1 h at 4 °C on a rotator followed by incubation with mouse anti-NKG2D (2 μg; clone 1D11, BioLegend) overnight at 4 °C on a rotator. Thereafter, protein G dynabeads (1.5 mg, Thermo) were added and incubated for an additional 3 h at 4 °C on a rotator. After recovery of the beads with a magnet, beads were washed three times for 3 min at 4 °C in 20 volume of ice-cold NP40 lysis buffer and then resuspended in 1 × NuPAGE LDS sample buffer (Invitrogen) containing 50 mM dithiothreitol (Sigma), and further incubated for 5 min at 95 °C for the elution of target protein from the protein G dynabeads. Western blot analysis of both the input (5% of the whole cell lysate) and the precipitated samples was performed using anti-Myc-tag (Cell Signaling, #2276) and anti-mouse IgG-HRP (Abcam, #ab131368) to visualize NK-CARs that were isolated by the immunoprecipitation with anti-NKG2D.

### Immunoblotting

Equal amounts of protein from each sample were resolved on an 8% Tris–HCl gel and subsequently transferred onto a PVDF membrane (Millipore) in transfer buffer (25 mM Tris, 192 mM glycine, 20% (v/v) methanol). The membranes were then blocked with 5% skim milk (Difco) in TBS-T (Tris-buffered saline containing 0.1% Tween-20) for 1 h and subsequently incubated with primary antibody and then with the HRP-conjugated secondary antibody. Blots were developed using SuperSignal West Pico (Thermo) and detected using LAS-4000 (Fujifilm). The following antibodies were used: pAkt-Ser473 (Cell Signaling, #9271), Akt (Cell Signaling, #9272), phospho-Erk1/2-Thr202/Tyr204 (Cell Signaling, #9101), Erk1/2 (Cell Signaling, #9272), and mouse anti-rabbit IgG-HRP (Santa Cruz, #sc-2357).

### Enzyme-Linked Immunosorbent Assay (ELISA)

The production levels of MIP-1α (R&D Systems), granzyme B (R&D Systems), and IFN-γ (Pierce) were determined by ELISA in cells that had been stimulated with beads coated with the rhCD19 Fc chimera or CD19-expressing REH cells. Briefly, beads for NK cell stimulation were prepared by incubating protein G Dynabeads (2 × 10^6^) with rhCD19 Fc chimera (0.2 μg) in PBS containing 1% FBS and 0.01% Tween-20 for 1 h at 4 °C. After washing three times with PBS containing 1% FBS and 0.01% Tween-20, the beads (2 × 10^6^) were incubated with rested NKL cells (0.2 × 10^6^) that can recapitulate receptor synergy [32] in 250 μL of RPMI, 5% FBS for 8 h at 37 °C. REH cells for NKL cell stimulation were prepared by incubation of 0.4 × 10^6^ REH cells with 0.2 × 10^6^ NKL cells in 250 μL of RPMI, 5% FBS for 8 h at 37 °C. The mixed cells were cultured in a 96-well v-bottom plate (Corning) and then the supernatants were subsequently analyzed.

### Caspase 3/7 assay

Active caspase 3/7 was detected using a Caspase 3/7 Activity Plate Reader Assay Kit, Red (STEMCELL Technologies, #100–0922). Target cells were co-incubated in 96-well flat-bottom plates (Corning) in triplicate at 20,000 cells/well with effector cells at the indicated E:T ratios for 1 h at 37 °C. The caspase 3/7 staining process after apoptosis induction was conducted in accordance with the manufacturer’s instructions. Briefly, after incubation of the target cells with effector cells, caspase 3/7 working solution containing caspase 3/7 substrate and assay buffer was added at a 1:1 ratio to the culture medium and incubated for 1 h in the dark at room temperature. Fluorescence was then detected using a Victor X4 multilabel plate reader (excitation at 540 nm, emission at 620 nm).

### NK cell cytotoxic degranulation and intracellular IFN-γ staining assay

The cytotoxic degranulation of NK cells was assessed by measuring the percent increase of CD107a expression on the cell surface. NK92 cells (0.1 × 10^6^) were incubated with an equal number of REH or Ramos cells for 2 h at 37 °C. After centrifugation, the cell pellets were resuspended in FACS buffer (DPBS with 1% FBS) and stained with anti-CD3-PerCP (clone SK7, BD Biosciences), anti-CD56-APC (NCAM16.2, BD Biosciences), and anti-CD107a/LAMP1-PE (clone H4A3, BD Biosciences) for 35 min in the dark at 4 °C. CD107a expression on the CD3^−^CD56^+^ NK92 cells was then analyzed by flow cytometry using FlowJo software (Tree Star). Cytokine production by the NK92 cells was determined by assessing the percent increase of intracellular expression of IFN-γ. In this assay, NK92 cells (0.1 × 10^6^) were stimulated with an equal number of the indicated target cells for 1 h at 37 °C. Thereafter, brefeldin A (GolgiPlug; BD Biosciences) and monensin (GolgiStop; BD Biosciences) were added, and the samples were incubated for an additional 5 h, for a total of 6 h. After this incubation, the cells were first stained with anti-CD3-PerCP and anti-CD56-APC for 35 min in the dark at 4 °C. After washing twice with FACS buffer, the NK92 cells were fixed and permeabilized with BD Cytofix/Cytoperm solution for 20 min in the dark at 4 °C. The cells were then washed twice with BD Perm/Wash buffer and stained with anti-IFN-γ-PE (clone 25723.11, BD Biosciences) overnight in the dark at 4 °C. Thereafter, the expression of IFN-γ in CD3^−^CD56^+^ NK cells was analyzed by flow cytometry.

### In vivo leukemia clearance assay

To assess whether CAR-NK cells specifically kill CD19-expressing target cells in vivo, we employed a leukemia clearance assay [44, 45] using CAR-NK92 cells introduced into immunodeficient mice. Briefly, 8–9-week-old female NOD/ShiLtJ-Rag2^*em1AMC*^Il2rg^*em1AMC*^ (NRGA) mice were purchased from JA BIO (Suwon, Gyeonggido, Korea) and injected intraperitoneally (i.p.) with CAR-NK92 cells (1 × 10^6^). REH cells were labeled with a carboxyfluorescein succinimidyl ester (CFSE) (1 µM; Invitrogen), while CD19 KO REH cells were labeled with FarRed (1 µM; Invitrogen). The cells were mixed at a 1:1 ratio (2 × 10^6^ cells of each cell type) and injected i.p. into NRGA mice. After 4 h, peritoneal lavage cells were collected using 10 mL syringe equipped with a 25 gauge needle and analyzed by flow cytometry, and the rejection of REH cells relative to CD19 KO REH cells was calculated as follows: ratio of residual cancer cells = residual REH cells (%) / residual CD19 KO REH cells (%).

### In vivo xenograft assay

8–9-week-old female NOD Cg-Prkdc^scid^ Il2rg^tm1Wjl^/SzJ (NSG) mice were purchased from JA BIO (Suwonsi, Gyeonggido, Korea), and REH cells (2 × 10^6^) were subcutaneously (s.c.) injected into the right flank of these animals. After the volume of the tumors reached 200–300 mm^3^, NK92, T-CAR3 NK92 or NK-CAR12 NK92 cells (5 × 10^6^) were injected intravenously (i.v.) on day 14, day 19, and day 26 for a total of three doses (*n* = 5 ~ 6 per group) as indicated in Fig. 7A. Subsequently, the tumor size was measured using digital calipers every other day as follows: tumor volume (V) = (L × W^2^) / 2 (L is tumor length and W is tumor width). Body weight changes were assessed on the same days as the tumor size measurements, given that the body weight loss of 20% is usually considered humane endpoint for euthanasia.

### Statistical analysis

All statistical analyses were conducted using GraphPad Prism version 8.0 software (GraphPad Software). Statistical comparisons among three or more groups was conducted using one- or two-way analysis of variance (ANOVA) followed by the Dunnett’s multiple comparison test. Statistical comparisons between two groups was performed using a two-tailed Student *t*-test or Mann–Whitney *U*-test. A *P*-value of less than 0.05 was considered to indicate statistical significance.

## Results

### Construction of NK-CARs that induce the synergistic activation of NK cells

**Fig. 1:**
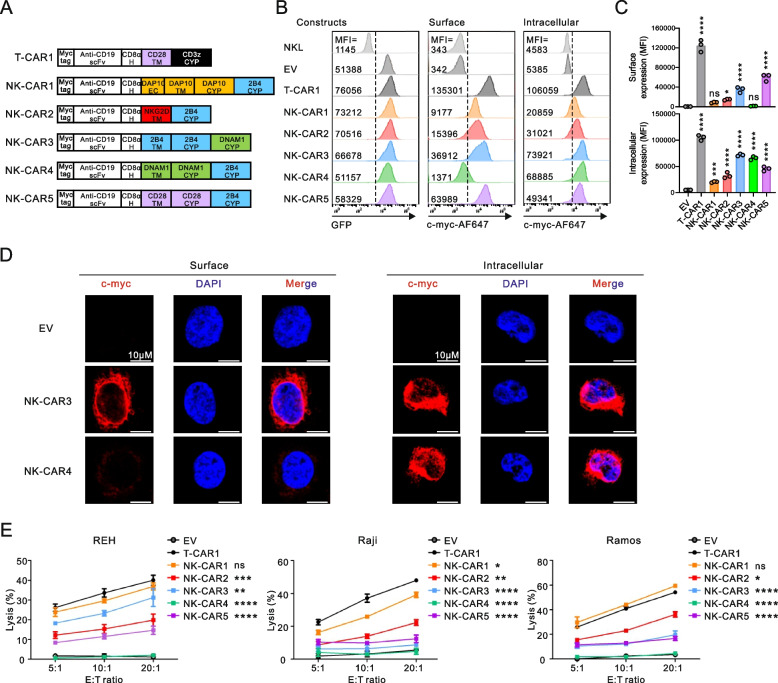
Construction of NK-CARs adopting the synergy of activating receptors. **A** Schematic diagram of the CAR constructs. All CARs harbor anti-CD19 scFv with a N-terminal myc-tag as an extracellular domain and the indicated combinations of signaling domains for the synergy of receptors 2B4 with NKG2D or DNAM-1. The hinge (H) and/or extracellular (EC), transmembrane (TM) and cytoplasmic (CYP) domains of the CARs are indicated. T-CAR1 was used as a positive control and contains a single CD3ζ chain activation domain. **B** Representative flow cytometry analysis showing the mean fluorescence intensity (MFI) of GFP (left panel) and surface CAR (middle panel) expression on CAR-transduced NKL cells. The right panel shows CAR localized inside the cells after permeabilization. **C** Summary graphs showing the MFI of surface (top) and intracellular CAR expression (bottom) in CAR-transduced NKL cells. **D** Confocal images of CAR NKL cells stained with anti-myc (red) and DAPI (blue). The left panel shows CAR expressed on the cell surface, and the right panel shows CAR accumulated inside the cells after permeabilization. Scale bars, 10 µm. **E** Specific lysis of REH, Raji, or Ramos cells by EV NKL or CAR NKL cells at the indicated E:T ratio. The levels of cytotoxicity against REH, Raji, or Ramos cells were measured using a 2 h europium release assay. Values represent the means ± SD. Data were analyzed using the one-way (**C**) or two-way ANOVA (**E**) with Dunnett’s multiple comparison test. ns, not significant; *P < 0.05; **P < 0.01; ***P < 0.001; ****P < 0.0001 relative to EV NKL cells (**C**) or T-CAR1 NKL cells (**E**). Data are representative of three independent experiments

NK cell activating receptors for natural cytotoxicity have a unique property to stimulate resting NK cells without cytokine pre-stimulation principally in combination with synergistic pairs of receptors [29, 31]. Among the receptor combinations that synergize to activate NK cells, we focused on the combinations of NKG2D and 2B4 or DNAM-1 and 2B4, which are well-characterized and frequently adopted for NK-CAR designs [18, 29]. To evaluate the functionality of NK-CARs, we used CD19 single-chain variable fragment (scFv) FMC63 with a Myc-tag as the extracellular (EC) domain to target CD19-positive cancer cells, which is linked to the CD8α hinge (H), transmembrane (TM) and cytoplasmic signaling domain (CYP). Diverse combinations of the TM and signaling domains were tested and compared to the first-generation T-CAR consisting of the CD28 TM and CD3ζ CYP (T-CAR1) for stepwise comparison of NK-CARs with T-CAR (Fig. 1A). We constructed and screened five different NK-CAR constructs designed to trigger synergistic activation of NK cells upon combination with the 2B4 activation domain. In humans, NKG2D couples to the DAP10 adaptor harboring a cytoplasmic ITT motif for intracellular signaling via an electrostatic interaction between the NKG2D TM and DAP10 TM [46]. Thus, NK-CARs with a DAP10 molecule (NK-CAR1) or NKG2D TM interacting with endogenous DAP10 (NK-CAR2) were constructed in combination with 2B4 CYP for NKG2D and 2B4 synergy. NK-CAR3 and NK-CAR4 were designed to combine the signaling domains of 2B4 and DNAM-1 in different orders for 2B4 and DNAM-1 synergy, given their signaling motifs in the cytoplasmic tails (ITSM for 2B4 and ITT-like for DNAM-1). NK-CAR5 was constructed to test whether CD28 signaling can substitute for NKG2D signaling when combined with 2B4 signaling, given the same ITT motif in DAP10 and CD28 CYP domain [34].

These five NK-CARs and T-CAR1 cloned into the pMX-retrovirus vector were transduced into NKL cells, a human NK cell line that can reproduce receptor synergy [32]. Due to the co-expression of GFP along with the cloned NK-CAR, transduced cells were sorted for a comparable expression of GFP (Fig. 1B, left). The NK-CARs except NK-CAR4 had a clear but variable surface expression, as assessed by anti-Myc antibody, which was detectably lower than that of T-CAR1 (Fig. 1B, middle and 1C). Despite its marginal surface expression, NK-CAR4 had a comparable intracellular expression to NK-CAR3 (Fig. 1B, right and 1C), indicating the dependence of CAR surface expression on the proper combination and order of TM and CYP domain. This finding was confirmed at a single cell level, as measured by the surface and intracellular levels of NK-CAR3 and NK-CAR4 in transduced NKL cells by confocal microscopy (Fig. 1D).

We next evaluated NK cell cytotoxicity against three different CD19-positive leukemia and lymphoma target cell types. The clear expression of CD19 on these target cells was observed with low expression evident on REH cells and high expression on Raji and Ramos cells (Supplementary Fig. 2). The results revealed that NK-CAR1 carrying the DAP10 adaptor and 2B4 CYP domain induced the strongest cytotoxicity irrespective of the target cell, and that this was comparable to or slightly lower than the level seen with T-CAR1 (Fig. 1E). Compared to empty vector (EV), NK-CAR4 did not improve NK cell cytotoxicity, correlating with its defective CAR surface expression. Hence, among the domain combinations tested, the DAP10 adaptor was more effective than NKG2D TM, DNAM-1 CYP, and CD28 CYP domain in combination with 2B4 CYP domain in inducing CAR-mediated cytotoxicity.

### Modification of the hinge (H), DAP10, and NKG2D domains to optimize NKG2D-mediated cytotoxicity

**Fig. 2:**
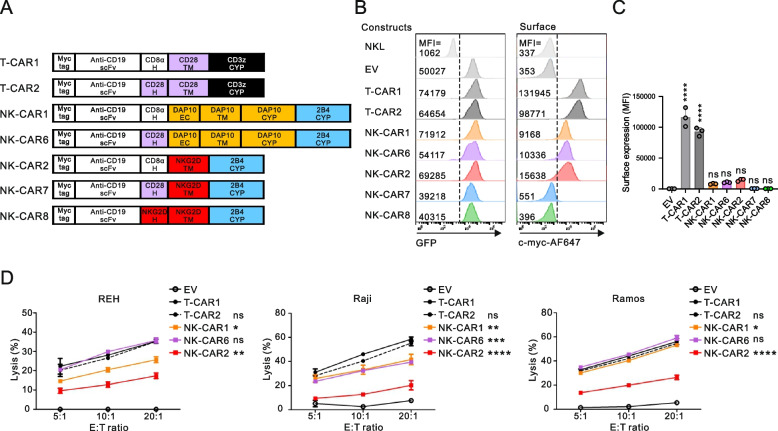
Comparison between the DAP10 and NKG2D TM domains for NKG2D-mediated activation. **A** Schematic diagram of CAR constructs with the indicated combinations of H and signaling domains for NKG2D and 2B4 receptor synergy. T-CAR1 and T-CAR2 contain a different H but share a single CD3ζ chain activation domain. **B** Representative flow cytometry analysis showing the MFI of GFP (left panel) and surface CAR (right panel) expression on the CAR-expressing NKL cells. **C** Summary graph showing the MFI of surface CAR expression in CAR-transduced NKL cells. **D** Specific lysis of REH, Raji, or Ramos cells by the indicated CAR-expressing NKL cells at the indicated E:T ratio. Values represent the means ± SD. Data were analyzed using the one-way (**C**) or two-way ANOVA (**D**) with Dunnett’s multiple comparison test. ns, not significant; *P < 0.05; **P < 0.01; ***P < 0.001; ****P < 0.0001 relative to EV NKL cells (**C**) or T-CAR1 NKL cells (**D**). Data are representative of three independent experiments

Having observed a more potent induction of NK cell cytotoxicity from the fusion of DAP10 and 2B4 CYP domain compared to other synergistic domain combinations, we focused on NK-CAR induction of NKG2D and 2B4 synergy and sought to engineer the CAR structures to improve the NK cell cytotoxicity response. The H domain, which connects the antigen (Ag)-binding and TM domain and provides flexibility to access the target Ag, can impact CAR expression, epitope recognition, and signal strength [18, 47]. We therefore replaced the CD8α H domain in NK-CARs with CD28 or NKG2D H domain and assessed CAR expression and NK cytotoxicity (Fig. 2A). Of note, a CD28 H domain (NK-CAR6) in replacement of CD8α H domain (NK-CAR1) improved the DAP10 (EC + TM + CYP) and 2B4 CYP-mediated cytotoxicity against CD19 + target cells, particularly REH cells, despite a similar CAR expression, which was comparable to the cytotoxic level seen with T-CAR1 (Fig. 2B-D). In comparison, a CD28 H (NK-CAR7) or NKG2D H domain (NK-CAR8) instead of CD8α H domain (NK-CAR2) abrogated the surface expression of CARs carrying the NKG2D TM and 2B4 CYP domain and the corresponding cytotoxicity (Fig. 2 B and C and data not shown). Moreover, the diminished CAR expression was not improved by insertion of the NKG2D CYP domain (NK-CAR2-1 and NK-CAR7-1) between NKG2D TM and 2B4 CYP domain (Supplementary Fig. 3). In contrast, there were no notable differences between the CD8α H (T-CAR1) and CD28 H domain (T-CAR2) in the expression and cytotoxicity of T-CARs. Again, we observed more effective CAR cytotoxicity mediated by the DAP10 adaptor and 2B4 CYP (NK-CAR6 and NK-CAR1) compared to NKG2D TM and 2B4 CYP (NK-CAR2), suggesting that the DAP10 adaptor rather than the NKG2D TM domain is a viable option for further CAR engineering.

**Fig. 3:**
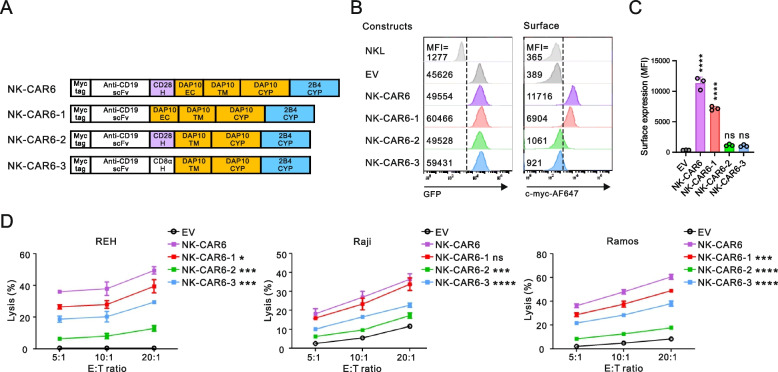
Coordinated effect of the CD28 hinge and DAP10 EC domain on the surface expression of NK-CARs. **A** Schematic diagram of CAR constructs with the indicated combinations of H and DAP10 domains with the 2B4 CYP domain. **B** Representative flow cytometry analysis showing the MFI of GFP (left panel) and surface CAR (right panel) expression on the CAR-expressing NKL cells. **C** Summary graph showing the MFI of surface CAR expression in CAR-transduced NKL cells. **D** Comparison of the lysis of REH, Raji, or Ramos cells by the indicated CAR-expressing NKL cells at the indicated E:T ratio. Values represent the means ± SD. Data were analyzed using the one-way (**C**) or two-way ANOVA (**D**) with Dunnett’s multiple comparison test. ns, not significant; *P < 0.05; ***P < 0.001; ****P < 0.0001 relative to EV NKL cells (**C**) or NK-CAR6 NKL cells (**D**). Data are representative of three independent experiments

Given the presence of an EC domain in the DAP10 adaptor, we next assessed whether this DAP10 EC domain could substitute for the extracellular CD28 H domain (Fig. 3A). CAR surface expression was impaired by the CD28 H domain deletion (NK-CAR6-1) but was almost abrogated by deleting the DAP10 EC domain (DAP10ΔEC) even if the CD28 H (NK-CAR6-2) or CD8α H domain (NK-CAR6-3) were present (Fig. 3 B and C). CAR-mediated cytotoxicity was also noticeably impaired by the deletion of CD28 H domain or DAP10 EC domain in particular (Fig. 3D), suggesting the requirement of a proper H domain for CAR-mediated cytotoxicity. Despite low CAR expression, the CD8α H domain (NK-CAR6-3) was superior to the CD28 H domain (NK-CAR6-2) in combination with DAP10 lacking an EC domain (DAP10ΔEC) in terms of CAR-mediated cytotoxicity. This suggested that CD28 H domain is favorably coupled to DAP10 EC domain, whereas CD8α H domain is suitable to compensate for DAP10ΔEC for CAR activation, as observed with NK-CAR1 vs NK-CAR6 (Fig. 2).

### Construction and optimization of NK-tailored CAR based on DAP10 and 2B4

**Fig. 4:**
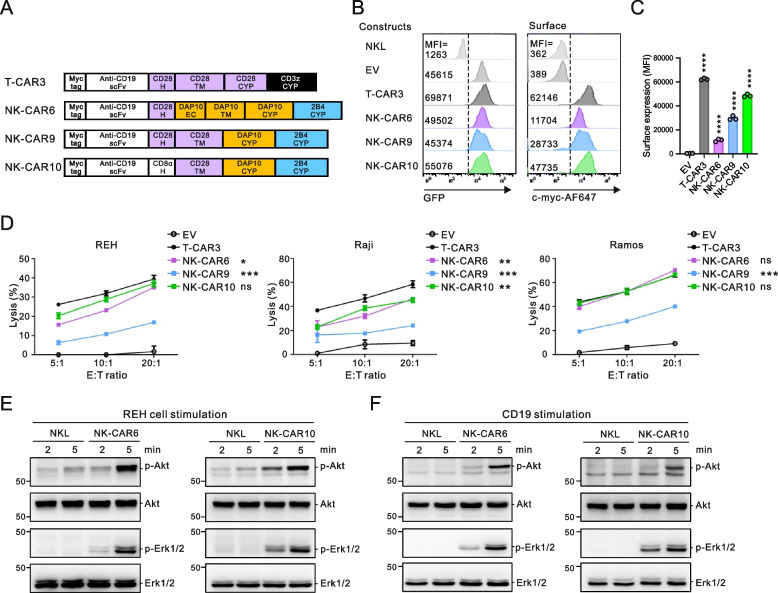
Optimization of the DAP10 domain for triggering NKG2D and 2B4 synergy. **A** Schematic diagram of CAR constructs with the indicated combinations of H, TM, and DAP10 domains with the 2B4 CYP domain. T-CAR3 harbors a combination of CD28 and CD3ζ chain signaling domains. **B** Representative flow cytometry analysis showing the MFI of GFP (left panel) and surface CAR (right panel) expression on the CAR-expressing NKL cells. **C** Summary graph presenting the MFI of surface CAR expression in CAR-transduced NKL cells. **D** Specific lysis of REH, Raji, or Ramos cells by the indicated CAR-expressing NKL cells at the indicated E:T ratio. **E** and **F** NKL or CAR NKL cells were stimulated with REH target cells (**E**) or beads coated with rhCD19 chimera (**F**) for the indicated times. Cell lysates were immunoblotted with antibodies to phospho-Akt at serine 473 (pS473), total Akt, phospho-Erk1 and 2, or total Erk1 and 2. Values represent the means ± SD. Data were analyzed using the one-way (**C**) or two-way ANOVA (**D**) with Dunnett’s multiple comparison test. ns, not significant; *P < 0.05; **P < 0.01; ***P < 0.001; ****P < 0.0001 relative to EV NKL cells (**C**) or T-CAR3 NKL cells (**D**). Data are representative of three independent experiments

The TM domain anchors the CAR to the cell membrane, connects the EC and intracellular signaling domains, and ultimately affects the expression level and stability of CAR, and its signal transduction potency [18, 47]. Given the low surface expression of NK-CAR6 relative to the T-CARs, we next investigated whether the type of TM influences CAR surface expression and cytotoxicity. Among others, CD28 TM is frequently used in NK-CARs [18], and T-CARs with CD28 TM (T-CAR1 and T-CAR2) have exhibited high levels of CAR surface expression (Fig. 2A-C). Accordingly, we constructed two NK-CARs with a different H domain (CD28 vs CD8α) linked to the common CD28 TM, in replacement of DAP10 EC and TM, and combined the DAP10 and 2B4 CYP signaling domains (NK-CAR9 and NK-CAR10) (Fig. 4A). A second-generation T-CAR consisting of CD28 TM and a combination of the CD28 and CD3ζ CYP domains (T-CAR3) was also constructed for comparison as a significant clinical impact has been reported for Axicabtagene ciloleucel (Yescata) which contains this same CAR structure [8]. Notably, the CAR surface expression level was demonstrably increased by the replacement of DAP10 EC and TM with CD28 TM, particularly in NK-CAR with CD8α H (NK-CAR10) compared to CD28 H domain (NK-CAR9) (Fig. 4 B and C). In addition, NK-CAR10 was superior to NK-CAR9 in its ability to kill CD19 + target cells, and was also more potent than NK-CAR6 or T-CAR1/2 but slightly less effective than T-CAR3 against REH cells (Fig. 4D and data not shown). We next tested whether the sequential order of CYP domains affected CAR expression and cytotoxicity, given the prior reports on NK-CARs driven by 2B4-DAP10 CYP domains as distinct from the DAP10-2B4 CYP domains currently used in NK-CAR10 [37, 38]. We observed a slight decrease in CAR expression but a prominent reduction in cytotoxicity for NK-CAR10-1 with 2B4-DAP10 CYP domains (Supplementary Fig. 4), indicating a better signaling performance of DAP10-2B4 CYP than 2B4-DAP10 CYP domains.

To determine the activation signals transmitted by NK-CARs, we next investigated the signaling molecules linked to NKG2D and 2B4 synergy. NKL cells bearing either NK-CAR6 or NK-CAR10 were stimulated by CD19 + REH cells (Fig. 4E) or by beads coated with recombinant human CD19 protein (Fig. 4F). It has been reported that the engagement of NKG2D but not 2B4 leads to Akt phosphorylation, and that their co-engagement results in synergistic Erk phosphorylation [32, 42]. As expected, the stimulation with REH cells induced an apparent phosphorylation of Akt and Erk in NKL cells expressing NK-CARs, and this was not observed in NKL cells without NK-CARs (Fig. 4E). Likewise, a similar phosphorylation result for Akt and Erk was observed upon the stimulation with CD19 protein coupled to beads (Fig. 4F), confirming that these activation signals are driven by the DAP10-2B4 CYP domains in NK-CARs.

### Enhanced CAR-mediated activation through the integration of CD3ζ signaling domain

**Fig. 5:**
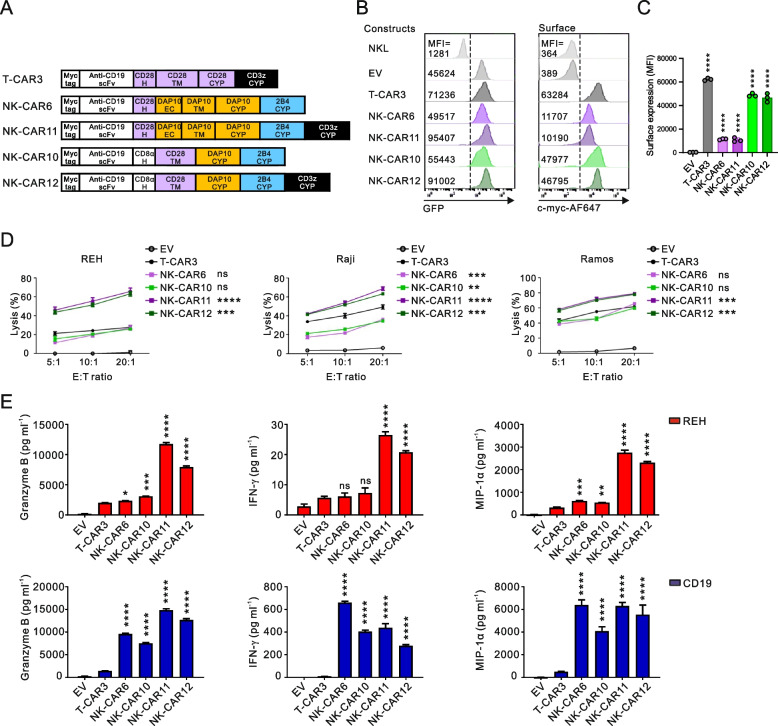
Superior CAR-mediated activation with the combinations of DAP10, 2B4, and CD3ζ signaling domains. **A** Schematic diagram of the CAR constructs with the indicated combinations of H, TM, and DAP10 domains with the 2B4 CYP domain with or without the addition of the CD3ζ CYP domain. T-CAR3 was used as a positive control. **B** Representative flow cytometry analysis showing the MFI of GFP (left panel) and surface CAR (right panel) expression on the CAR-expressing NKL cells. **C** Summary graph showing the MFI of surface CAR expression in CAR-transduced NKL cells. **D** Specific lysis of REH, Raji, or Ramos cells by EV NKL or CAR NKL cells at the indicated E:T ratio. **E** Different CAR NKL cells were stimulated with REH target cells (top) or beads coated with rhCD19 chimera (bottom) for 8 h. Granzyme B, IFN-γ, and MIP-1α in the culture supernatants were measured by ELISA. Values represent the means ± SD. Data were analyzed using the one-way (**C**, **E**) or two-way ANOVA (**D**) with Dunnett’s multiple comparison test. ns, not significant; *P < 0.05; **P < 0.01; ***P < 0.001; ****P < 0.0001 relative to EV NKL cells (**C**) or T-CAR3 NKL cells (**D**, **E**). Data are representative of three independent experiments

CD3ζ signaling domain is frequently used in CAR design and is common to TCR complex in T cells and CD16 in NK cells [18, 29]. Moreover, CD16 cooperates with other NK cell receptors including NKG2D and 2B4 to augment NK cell cytotoxicity [31, 48]. Thus, to further improve the antitumor activity of NK-CARs, we attached a CD3ζ CYP domain next to the DAP10-2B4 CYP domains common to NK-CAR6 (NK-CAR11) and NK-CAR10 (NK-CAR12) (Fig. 5A). Despite the retention of the CAR expression level, the addition of CD3ζ CYP domain to the DAP10-2B4 CYP domains remarkably enhanced the CAR-mediated cytotoxicity (NK-CAR11 and NK-CAR12) to a level that was even greater than that of T-CAR3 against all of the CD19 + target cells tested (Fig. 5B-D). Consistently, the cytotoxic release of granzyme B was prominently increased by NK-CAR11 and NK-CAR12 compared to T-CAR3 and other NK-CARs following stimulation with REH cells and CD19 protein coupled to beads (Fig. 5E, left). Similarly, the production of cytokine IFN-γ and chemokine MIP-1α was substantially increased by NK-CAR11 and NK-CAR12 upon stimulation with REH cells but was comparable upon the stimulation of NK-CARs with CD19 protein coupled to beads (Fig. 5E, middle and right). Hence, NK-CARs that combined DAP10, 2B4, and CD3ζ CYP domains could induce greater effector functions of NK cells than conventional T-CAR3.

Compared to NK-CAR12, NK-CAR11 contained a DAP10 TM domain that is capable of interacting with the TM domain of NKG2D receptor (Supplementary Fig. 5A) and thereby might affect NK cell function [46]. To assess such a possibility, NKG2D receptor was immunoprecipitated from the lysates of NKL or NKL cells bearing NK-CAR11 and NK-CAR12 and probed with an antibody to Myc-tag for CAR detection. NK-CAR11, but not NK-CAR12, showed a clear association with endogenous NKG2D receptor and triggered NK cell cytotoxicity against K562 cells lacking CD19 expression (Supplementary Fig. 5B and 5C). Thus, unlike NK-CAR11, NK-CAR12 was found to be tightly regulated to trigger target Ag-restricted cytotoxicity of NK cells despite their similar potency in NK cell activation.

### A potent cytotoxicity mediated by NK-CAR12 with DAP10-2B4-CD3ζ signaling domains

**Fig. 6:**
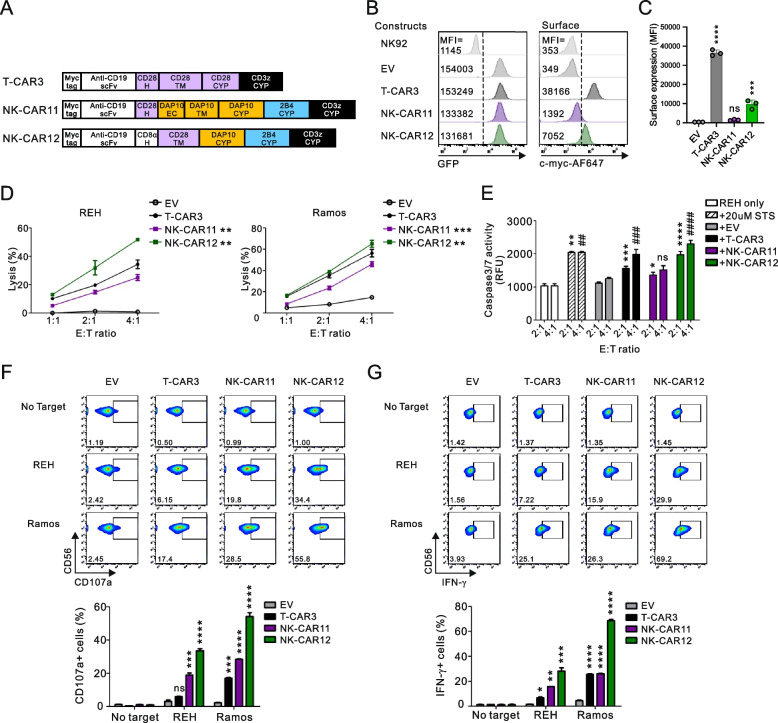
Superior expression and antitumor activity of NK-CAR12 in NK cells. **A** Schematic diagram of the final CAR constructs with CD28 (H), DAP10 (EC + TM + CYP), 2B4 (CYP), and CD3ζ (CYP) for NK-CAR11 and CD8α (H), CD28 (TM), DAP10 (CYP), 2B4 (CYP), and CD3ζ (CYP) for NK-CAR12. T-CAR3 was used as a positive control. **B** Representative flow cytometry analysis showing the MFI of GFP (left panel) and surface CAR (right panel) expression on CAR NK92 cells. **C** Summary graph showing the MFI of surface CAR expression in CAR-transduced NK92 cells. **D** Specific lysis of REH or Ramos cells by the indicated CAR-expressing NK92 cells at the indicated E:T ratio. The cytotoxicity was measured using a 30 min europium release assay. **E** REH cells were treated with the indicated CAR NK92 cells for 1 h. Active caspase 3/7 in the REH cell lysates was detected using the caspase 3/7 activity assay kit. 20 µM staurosporine (STS) was used as a positive control. **F** and **G** Representative FACS profiles showing the percentage of CD107a^+^ CAR NK92 cells after 2 h of stimulation (**F**) and the percentage of IFN-γ^+^ CAR NK92 cells after 6 h of stimulation (**G**) with different target cells (top). Summary graphs showing the percentage of CD107a^+^ and IFN-γ^+^ CAR NK92 cells (bottom). Values represent the means ± SD. Data were analyzed using the one-way (**C**, **E**–**G**) or two-way ANOVA (**D**) with Dunnett’s multiple comparison test. ns, not significant; *P < 0.05; **P < 0.01; ***P < 0.001; ****P < 0.0001 relative to EV NK92 cells (**C**, **E** at the E:T ratio of 2:1, **F**, **G**) or T-CAR3 NK92 cells (**D**). ^##^*P* < 0.01; ^###^*P* < 0.001; ^####^*P* < 0.0001 relative to EV NK92 cells (**E** at the E:T ratio of 4:1). Data are representative of three independent experiments

To validate the results obtained with the NKL cells, we used NK92 cells, a representative human NK cell line currently used in clinical trials [25, 49]. We thus expressed NK-CAR11 or NK-CAR12 in NK92 cells, based on their potent cytotoxicity in NKL cells, and compared their potency with T-CAR3 (Fig. 6A). Although lower than the T-CAR3, NK-CAR12 induced a higher CAR expression level than NK-CAR11 (Fig. 6 B and C). Accordingly, NK-CAR12 was superior to NK-CAR11 in its capacity to kill CD19 + target cells, particularly REH cells, and this was also higher than T-CAR3 (Fig. 6D). The superior cytotoxicity mediated by NK-CAR12 over NK-CAR11 was confirmed by assessing apoptotic caspase 3/7 activity in REH cells following their co-incubation with CAR-expressing NK92 cells (Fig. 6E). Correlating with these results, the expression level of CD107a, a generally used marker for cytotoxic degranulation, on NK cells was highest with NK-CAR12, followed in decreasing order by NK-CAR11 and T-CAR3 upon stimulation with REH and Ramos cells (Fig. 6F). A similar hierarchy was observed for IFN-γ expression by stimulated CAR-NK92 cells in the order of NK-CAR12, NK-CAR11, and T-CAR3 (Fig. 6G).

Having observed a potent cytotoxicity mediated by NK-CARs and given that the loss of targeted Ag from cancer cells is a major barrier to CAR therapy, we next assessed whether NK-CARs would be effective in eliminating target cells with low Ag levels. To this end, REH cells were sorted according to their CD19 expression (i.e. high, intermediate, and low) (Supplementary Fig. 6A). Among others, NK-CAR12 efficiently killed REH cells even with a low level of CD19 expression (Supplementary Fig. 6B), further indicating the effectiveness of NK-tailored CAR for triggering NK cell cytotoxicity.

Next, we investigated the potency of NK-CAR12 in comparison with T-CAR3 in primary human NK cells. Primary NK cells that were expanded by using feeder cells and then transduced with NK-CAR12 or T-CAR3 were used as effector cells. CAR-expressing NK cells were identified by the co-expression of GFP along with the cloned NK-CAR. GFP-positive primary NK cells bearing NK-CAR12 was more potent than the primary NK cells with T-CAR3 in their capacity to degranulate in response to CD19 + target cells, particularly REH cells (Supplementary Fig. 7). In comparison, cytotoxic degranulation was comparable between their respective GFP-negative primary NK cells as expected.

### Potent antitumor effects of NK-CAR12 in vivo

We next addressed whether NK-CAR12 also exhibits potent cytotoxicity against REH cells in vivo. To this end, we employed a lymphoma clearance assay [44, 45] modified to compare the clearance of cancer cells with distinct susceptibilities to CAR-NK cells in the same mice. The CRISPR/Cas9 system was used to produce a series of *CD19* deletion mutants in REH cells and generate CD19 knockout (KO) REH cells resistant to CAR-NK cells. The two guide RNA sequences in exon 1 and exon 2 of *CD19* were targeted to delete CD19 protein (Supplementary Fig. 8A). After screening of CD19 expression on REH cells, the corresponding CD19 KO REH cell clone was selected (Supplementary Fig. 8B). Accordingly, these CD19 KO cells, but not the parental REH cells, were highly resistant to NK92 cells bearing CARs including NK-CAR12 (Supplementary Fig. 8C). Thereafter, equal numbers of wild-type and CD19 KO REH cells were co-injected intraperitoneally (i.p.) into immune-deficient NRGA mice lacking NK cells after an i.p. injection of NK92 cells expressing NK-CAR12 or T-CAR3 at an E:T ratio of 2:1 (Supplementary Fig. 8D). For their identification, REH cells and CD19 KO REH cells were labeled with CellTrace CFSE and FarRed, respectively. Subsequent analysis of peritoneal exudates by flow cytometry revealed a significant increase in the clearance of REH cells relative to CD19 KO REH cells by CAR-NK92 cells, particularly NK-CAR12-expressing NK92 cells (Supplementary Fig. 8E).

**Fig. 7:**
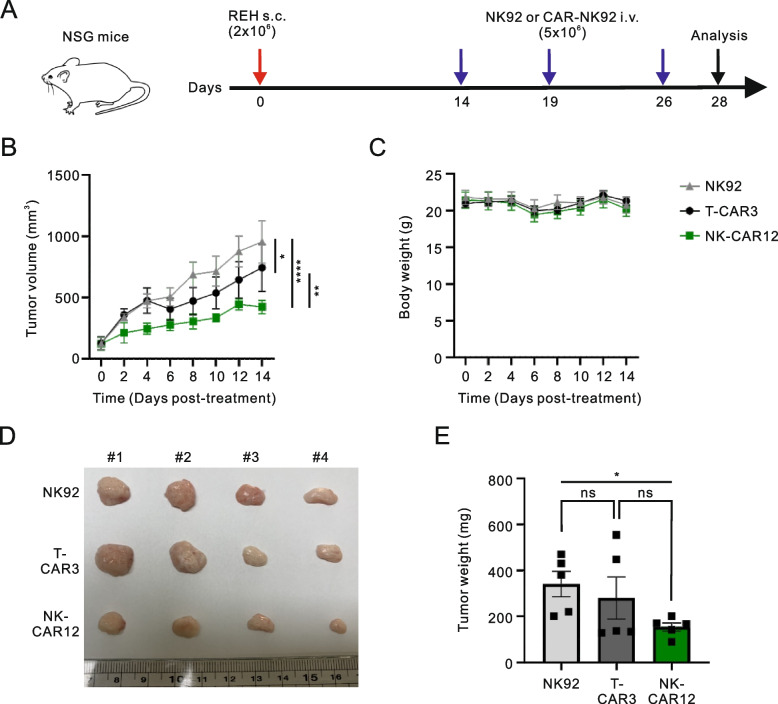
NK-CAR12-bearing NK cells exhibit potent antitumor effects in NSG mice. **A** Experimental scheme. NSG mice (*n* = 5 each group) were subcutaneously injected with 2 × 10^6^ REH cells. On day 14, 5 × 10.^6^ NK92, T-CAR3 NK92, or NK-CAR12 NK92 cells were injected into the tail vein three times periodically. After a total of 28 days, the mice were euthanized and analyzed. **B** Analysis of tumor volume to quantitate tumor growth inhibition. **C** Measurement of mouse body weights after treatment with the indicated CAR NK92 cells. **D** Photographs of representative tumors dissected from the mice after study termination. **E** Analysis of tumor weights after study termination. Values represent the means ± SEM; each dot represents an individual mouse. Data were analyzed using the two-way ANOVA with Dunnett’s multiple comparison test (**B**) or Mann–Whitney *U*-test (**E**). ns, not significant; *P < 0.05; **P < 0.01; ****P < 0.0001

To then verify these antitumor effects of NK-CAR12 in a different mouse model, immune-deficient NSG mice were implanted with REH cells via subcutaneous (s.c.) injection and then intravenously (i.v.) injected with NK92 cells or CAR-NK92 cells on day 14 for a total of three doses (Fig. 7A). The growth of tumors was significantly decreased by the administration of CAR-NK92 cells, particularly NK-CAR12-bearing NK92 cells (Fig. 7B), without a significant effect on body weight (Fig. 7C). The potent antitumor effects of NK-CAR12 compared to T-CAR3 were further validated by assessing the size and weight of tumors excised from the tumor-burdened mice at the end of the experiment (Fig. 7 D and E). Overall, these results indicated a potent antitumor effect of NK-CAR12 and suggested that the engineering of NK-tailored CAR incorporating NK-specific signaling domains is a viable strategy to improve the antitumor potency of CAR-NK cells.

## Discussion

The choice of CAR components such as H, TM, and signaling domains prominently affects the functional outcome of the CAR-bearing cells. As these studies were conducted mainly for T cells, and only few combinations of NK cell activation domains were tested for CAR design, there is a vital need for structured analysis of the optimal combination of activation domains for CAR-NK cells. In this study, we utilized a stepwise approach to modifying CAR components (Table 1) and observed that NK-tailored CAR, particularly NK-CAR12 incorporating the signaling domains of DAP10, 2B4, and CD3ζ coupled to CD8α H and CD28 TM, outperforms the conventional T-CAR currently used to treat hematological malignancies. This NK-CAR that combines distinct signaling modules triggered stronger cytotoxic activity than T-CAR against CD19-expressing lymphoma in vitro and in vivo. Moreover, NK cells expressing this NK-CAR could efficiently kill lymphoma cells with a low level of CD19 expression. These findings support the notion that optimizing domain combinations and tailored adaptation of the CAR construct for NK cells are promising strategies for improving NK cell-based immunotherapies.

Among the large repertoire of NK cell activating receptors and adaptor proteins, we focused on combination of signaling domains from activating receptors that synergize each other to improve CAR-mediated cytotoxicity. In previous studies, the NK92 cell line was used in the screening of NK-CARs that incorporate combinations of activation domains among NKG2D, 2B4, and DNAM-1 [38, 39, 50, 51]. In comparison, we used the NKL cell line to evaluate the functionality of the NK-CARs designed for receptor synergy since NKL cells, but not NK92, NK3.3, or YTS cells, are a suitable surrogate for resting NK cells that can reproduce synergistic activation upon NKG2D and 2B4 co-engagement [32]. We found via a series of systematic comparisons that combinations of signaling domains for NKG2D and 2B4 synergy were superior to those for DNAM-1 and 2B4 synergy. Moreover, use of the DAP10 adaptor, particularly DAP10 CYP domain, outperformed NKG2D TM, DNAM-1 CYP, and CD28 CYP domain to trigger CAR-mediated cytotoxicity in combination with the 2B4 CYP domain. Interestingly, one NKG2D TM domain provides an interaction site for two DAP10 adaptor proteins recruiting individual PI3K and Grb2/Vav1 [33, 46] but was less effective than a DAP10 CYP domain in NK-CAR construct for cytotoxic activation as opposed to our expectation (Fig. 1E). For provoking synergy, NKG2D and 2B4 need to be segregated at NK cell immune synapses with 2B4 preferentially in the center and NKG2D in the periphery [52]. Thus, we speculate that this spatial configuration may affect proper binding of NKG2D TM to DAP10 and activation of downstream signaling pathways when directly linked to 2B4 CYP domain. In this regard, it merits further investigation including the assessment of PI3K pathway. In addition, a sequential assembly of DAP10 and 2B4 signaling domains (DAP10-2B4) was superior to their inverse assembly (2B4-DAP10) for generating CAR-mediated cytotoxicity. Collectively, these results have highlighted the utility of a proper combination and order of signaling domains to improve CAR signaling responses for NK cells. This notion is compatible with the finding that the number, type, and order of costimulatory domains can likely dictate the structural compatibility with H and TM domain and affect CAR-T functionality [47, 53].

To further enhance the antitumor activity of NK-CARs driven by ITAM-independent DAP10 and 2B4 signaling domains, ITAM-containing CD3ζ signaling domain was added. CD3ζ chain contains three ITAMs in its cytoplasmic tail and associates with CD16 and NKp46 in NK cells [29, 54]. Moreover, CD16 cooperates with NKG2D and 2B4 but not with NKp46 to efficiently augment NK cell activation [31]. Signaling through ITAMs recruits Syk/ZAP70 tyrosine kinase and transmits activation signals, similar to T cell activation via TCR-CD3 complex [29, 30]. NKG2D signals through its association with DAP10 harboring ITT motif [33, 34, 36], whereas 2B4 transmits Vav1-dependent activation signals via four cytoplasmic ITSMs [35]. Given the synergistic combination of activating receptors with different signaling modules [29], we speculate that NK-CARs incorporating an additional CD3ζ signaling domain integrate distinct and complementary activation signals and can possibly optimize the antitumor activity through both ITAM-dependent and -independent signaling pathways. The resulting NK-CAR12 comprising DAP10, 2B4, and CD3ζ signaling domains induced the cytolysis of CD19-expressing target cells more potently, both in vitro and in vivo, than conventional T-CAR3 driven by CD28 and CD3ζ signaling domains. Moreover, given the sporadic expression of ligands for NKG2D on normal tissues and cells [55–57], NK-CAR12 may also pose less risk of off-target toxicity, a safety concern of unintended Ag attack in CAR-T therapy, than NK-CAR11 with DAP10 TM recruiting endogenous NKG2D.

In addition to signaling domains, the extracellular H and TM domains significantly influence CAR expression, signal transduction, and antitumor activity [18, 53]. We observed an abrogation of surface expression of CARs carrying NKG2D TM and 2B4 CYP domain following a replacement of CD8α H domain (NK-CAR2) with either CD28 H (NK-CAR7) or NKG2D H domain (NK-CAR8) (Fig. 2 B and C). Moreover, the surface expression of CAR carrying DAP10 adaptor and 2B4 CYP domain was impaired by the deletion of CD28 H domain (NK-CAR6-1) and abrogated by the deletion of DAP10 EC domain (DAP10ΔEC), irrespective of the presence of CD28 H (NK-CAR6-2) or CD8α H domain (NK-CAR6-3) (Fig. 3 B and C). In comparison, CAR surface expression was clearly elevated by replacing DAP10 EC and TM with CD28 TM, particularly in NK-CAR coupled to CD8α H (NK-CAR10) rather than CD28 H domain (NK-CAR9) (Fig. 4 B and C). In contrast, the addition of CD3ζ CYP signaling domain to DAP10-2B4 CYP domains did not affect CAR expression but prominently enhanced the levels of CAR-mediated cytotoxicity (NK-CAR11 and NK-CAR12) (Fig. 5B-D). These results suggest a significant effect of H and TM domains rather than signaling domain on CAR expression and the requirement of their optimization for NK-tailored CAR design.

Although we observed the most potent cytotoxicity induced by NK-CAR12 comprising two costimulatory domains (DAP10-2B4) and CD3ζ CYP domain, T-CAR3 with a single CD28 costimulatory domain linked to CD3ζ CYP domain was quite performing well in producing antitumor cytotoxicity. In support, a recent study showed that CD28 signaling domain is superior to other signaling domains from various costimulatory molecules including DAP10, DAP12, and 4-1BB in enhancing the potency of CAR construct via a conserved LCK/CD3ζ/ZAP70 signaling axis [58]. Given the efficacy of 2B4 costimulation in CAR-NK cells [51, 59] and the presence of ITT motif in both DAP10 and CD28, it would be interesting to test whether the inclusion of 2B4 CYP domain in T-CAR3 further improves the antitumor cytotoxicity as did in NK-CAR12. While CD28 rather than DAP10 costimulation is suitable to augment CD3ζ-mediated activation [58], our results of stronger cytotoxicity with NK-CAR1 than NK-CAR5 (Fig. 1E) led to the speculation that 2B4 CYP domain appears better coupled to DAP10 than CD28 CYP domain for receptor synergy. However, given the impact of distinct H, TM, and signaling domains (i.e. type and order) on CAR-T functionality, future systematic studies would be required to compare DAP10 and CD28 costimulation in NK-CARs carrying 2B4 and CD3ζ signaling domains. Moreover, while DAP12 and 4-1BB have been also used in NK-CAR designs [18], their contribution to NK-CARs was not evaluated in this study primarily focusing on NK-specific receptor synergy. DAP12 is associated with NK activating receptors including NKp44 and NKG2C and has been used in place of CD3ζ signaling domain for CAR-NK cells [49, 60, 61]. 4-1BB costimulation in CAR-T cells preferentially induces memory-associated genes and sustained antitumor activity [62] but its contribution to CAR-NK cells is not well established. In this regard, it merits further investigation on the extent to which these costimulation improve CAR-NK cell responses when properly incorporated into NK-CAR constructs.

In addition to optimizing NK-tailored CAR construct, further engineering of CAR-NK cells with on-board cytokines (e.g., IL-15) leading to enhanced in vivo persistence and activation holds promise to pave the way for improving clinical efficacy [63, 64]. Fourth-generation CAR-T cells engineered to express cytokines in soluble or membrane-bound form were validated to support long-term persistence and enhance antitumor activities [65, 66]. This therapeutic option is particularly relevant for NK cells, given their short half-life post-infusion as a primary challenge in NK cell therapy [67]. Armoring with transgenic IL-15 enhance the function, long-term persistence, and clinical efficacy of CAR-NK cells in patients with lymphoid malignancies via a support for metabolic fitness [63, 64, 68]. In this regard, the rational inclusion of supplemental cytokines (e.g., IL-15, IL-21) into CAR constructs may offer a promising treatment strategy for sustaining antitumor activity of CAR-NK cells and improving clinical outcome.

## Conclusions

In summary, these studies demonstrate the utility of complementary NK-specific activation domains to create NK-tailored CAR for improving the antitumor activity of NK cells. Our present results also provide significant insights into the rational design of optimized NK-CARs via a stepwise approach to modify the combination and order of the H and TM domains as well as signaling domains. The use of ITAM-independent signaling domains that synergize each other (i.e. DAP10 and 2B4 CYP domains) mediated complementation with the conventional ITAM-dependent CD3ζ signaling domain to enhance the function of CAR-NK cells. The large repertoire of NK cell activating receptors and adaptor proteins provides a pool of signaling domains to endow CAR-NK cells with enhanced functionalities. In this context, further systematic comparisons across different signaling domains along with their adaptation to appropriate H and TM domains is merited to enable the continued refinement of CAR constructs to optimize CAR-NK cell therapies. Finally, we anticipate that continuing advances in CAR design will enable the introduction of molecular payloads such as cytokine armoring in addition to NK-tailored CARs and thereby expand the therapeutic benefits of CAR-NK cells.

## Supplementary Information

Supplementary Material 1

## Data Availability

All data needed to evaluate the conclusions in the paper are present in the paper and/or the Supplementary Materials. Additional data related to this paper may be available from the corresponding author upon reasonable request.

## Abbreviations

CAR: Chimeric antigen receptor
EC: Extracellular
H: Hinge
TM: Transmembrane
NK: Natural killer
scFv: Single-chain variable fragment
MHC: Major histocompatibility complex
CRS: Cytokine release syndrome
TCR: T-cell receptor
GvHD: Graft-versus-host disease
iPSC: Induced pluripotent stem cells
NCR: Natural cytotoxicity receptor
ITAM: Immunoreceptor tyrosine-based activation motif
ITT: Immunoglobulin tyrosine tail
ITSM: Immunoreceptor tyrosine-based switch motif
RPMI-1640: Roswell Park Memorial Institute-1640
FBS: Fetal bovine serum
rIL-2: Recombinant IL-2
MEM-α: Minimum Essential Medium-α
ALL: Acute lymphocytic leukemia
IMDM: Iscove's Modified Dulbecco's Medium
DMEM: Dulbecco's Modified Eagle Medium
SP: Signal peptide
CYP: Cytoplasmic signaling domain
OE-PCR: Overlap extension PCR
IRES: Internal ribosome entry site
EGFP: Enhanced green fluorescent protein
SCGM: Stem Cell Growth Medium
RNP: Ribonucleoprotein
KO: Knock-out
ELISA: Enzyme-Linked Immunosorbent Assay
NRGA: NOD/ShiLtJ-Rag2^*em1AMC*^Il2rg^*em1AMC*^
i.p.: Intraperitoneally
CFSE: Carboxyfluorescein succinimidyl ester
NSG: NOD Cg-Prkdc^scid^ Il2rg^tm1Wjl^/SzJ
s.c.: Subcutaneously
i.v.: Intravenously
EV: Empty vector
