# Supplementary material for: A chimeric antigen receptor tailored to integrate complementary activation signals potentiates the antitumor activity of NK cells
Source: J Exp Clin Cancer Res. 2025 Mar 6;44:86. doi: 10.1186/s13046-025-03351-5 (PMC11884141; doi:10.1186/s13046-025-03351-5)
Supplement: Supplementary file 1 — Supplementary Material 1 [file 13046_2025_3351_MOESM1_ESM.docx]

**Supplementary Table 1. List of amino acid sequences used for CAR domains**

| Name | Domain | Amino acid sequence | |
| --- | --- | --- | --- |
| CD8α | H | NM_001145873.1, a.a.: 138-182 | TTTPAPRPPTPAPTIASQPLSLRPEACRPAAGGAVHTRGLDFACD |
| CD28 | H | NM_006139.4, a.a.: 114-152 | IEVMYPPPYLDNEKSNGTIIHVKGKHLCPSPLFPGPSKP |
| DAP10 | EC | NM_014266.4, a.a.: 19-48 | QTTPGERSSLPAFYPGTSGSCSGCGSLSLP |
| NKG2D | H | NM_007360.4, a.a.:90-80 | PIQVEQNFLSN |
| CD28 | TM | NM_006139.4, a.a.: 153-179 | FWVLVVVGGVLACYSLLVTVAFIIFWV |
| DAP10 | TM | NM_014266.4, a.a.: 49-69 | LLAGLVAADAVASLLIVGAVF |
| NKG2D | TM | NM_007360.4, a.a.: 79-48 | LFVASWITVMIIFRIGMAVAIFCCFFFPSANE |
| 2B4 | TM | NM_016382.4, a.a.: 222-245 | FWPFLVIIVILSALFLGTLACFCV |
| DNAM-1 | TM | NM_001303619.2, a.a.: 93-120 | QYTLFVAGGTVLLLLFVISITTIIVIFL |
| CD28 | CYP | NM_006139.4, a.a.: 180-220 | RSKRSRLLHSDYMNMTPRRPGPTRKHYQPYAPPRDFAAYRS |
| DAP10 | CYP | NM_014266.4, a.a.: 70-93 | LCARPRRSPAQEDGKVYINMPGRG |
| NKG2D | CYP | NM_007360.4, a.a.: 47-1 | RCKSKVVPCRQKQWRTSFDSKKLDLNYNHFESMEWSHRSRRGRIWGM |
| 2B4 | CYP | NM_016382.4, a.a.: 246-365 | WRRKRKEKQSETSPKEFLTIYEDVKDLKTRRNHEQEQTFPGGGSTIYSMIQSQSSAPTSQEPAYTLYSLIQPSRKSGSRKRNHSPSFNSTIYEVIGKSQPKAQNPARLSRKELENFDVYS |
| DNAM-1 | CYP | NM_001303619.2, a.a.: 121-181 | NRRRRRERRDLFTESWDTQKAPNNYRSPISTSQPTNQSMDDTREDIYVNYPTFSRRPKTRV |
| CD3ζ | CYP | NM_000734.3, a.a.:52-163 | RVKFSRSADAPAYQQGQNQLYNELNLGRREEYDVLDKRRGRDPEMGGKPRRKNPQEGLYNELQKDKMAEAYSEIGMKGERRRGKGHDGLYQGLSTATKDTYDALHMQALPPR |

Abbreviations: H, Hinge; EC, Extracellular domain; TM, Transmembrane domain; CYP, Cytoplasmic signaling domain

**Supplementary Figures**


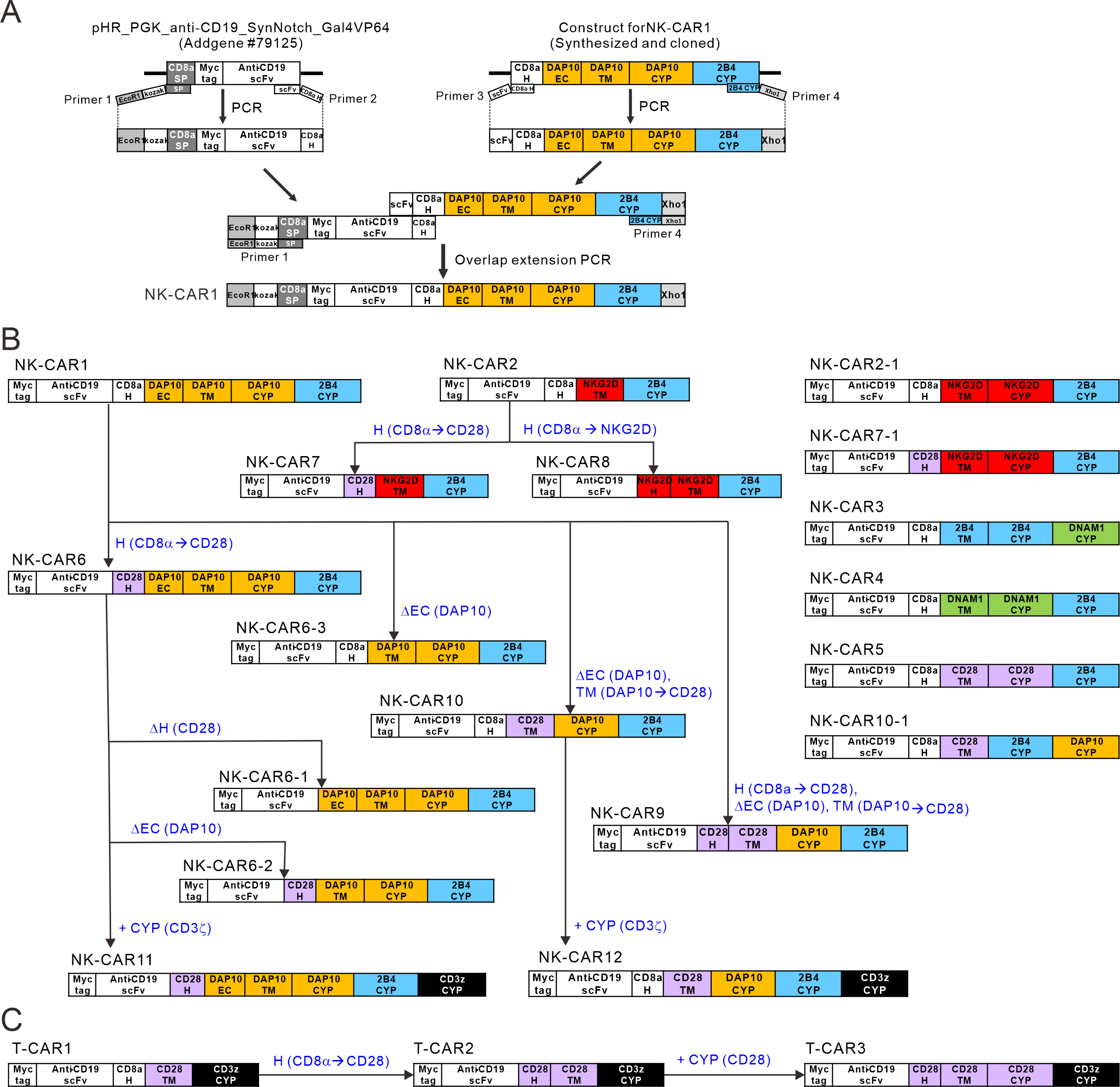


## **Supplemental Figure 1. Schematic diagram of the CAR cloning strategy and generation of NK-CAR and T-CAR constructs.**

(**A**) Cloning strategy for NK-CAR1 by overlap extension PCR. A PCR fragment encoding the CD8α signal peptide (SP), myc tag, and anti-CD19 scFv sequences was amplified from the pHR_PGK_anti-CD19_SynNotch_Gal4VP64 plasmid (Addgene #79125) using the primer 1 and primer 2 with a 20 bp overlapping DNA sequence with the CD8α SP and anti-CD19 scFv, respectively. Another PCR product containing the CD8α (H), DAP10 (EC+TM+CYP), and 2B4 CYP sequences was amplified from the plasmid synthesized for NK-CAR1 using the primer 3 and primer 4 with a 20 bp overlapping DNA sequence with the CD8α (H) and 2B4 CYP, respectively. These two PCR products were combined, and the entire NK-CAR1 was generated using the primer 1 and primer 4 containing EcoR1 and Xho1 restriction site, respectively. The DNA encoding the full-length NK-CAR1 was cloned into the EcoR1 and Xho1 sites of the pMXs-IRES-EGFP retroviral vector to create the anti-CD19-NK-CAR1 plasmid.

(**B**) Schematic representation for the stepwise generation of NK-CARs. The constructs encoding the H and/or EC, TM, and CYP domains for NK-CAR1, NK-CAR2, NK-CAR2-1, NK-CAR7-1, NK-CAR3, NK-CAR4, NK-CAR5, and NK-CAR10-1 were synthesized, fused to CD8α SP, myc tag, and anti-CD19 scFv by overlap extension (OE)-PCR as described in (A), and then used for subsequent NK-CAR modifications. The modifications are indicated by arrows and detailed in blue.

(**C**) Schematic illustration for the generation of T-CARs. The sequence encoding the CD8α H, CD28 TM, and CD3ζ CYP domains of T-CAR1 was synthesized, fused to CD8α SP, myc tag, and anti-CD19 scFv by OE-PCR as described in (A), and then used for the generation of T-CAR2 and T-CAR3. The modifications are indicated by arrows and detailed in blue.


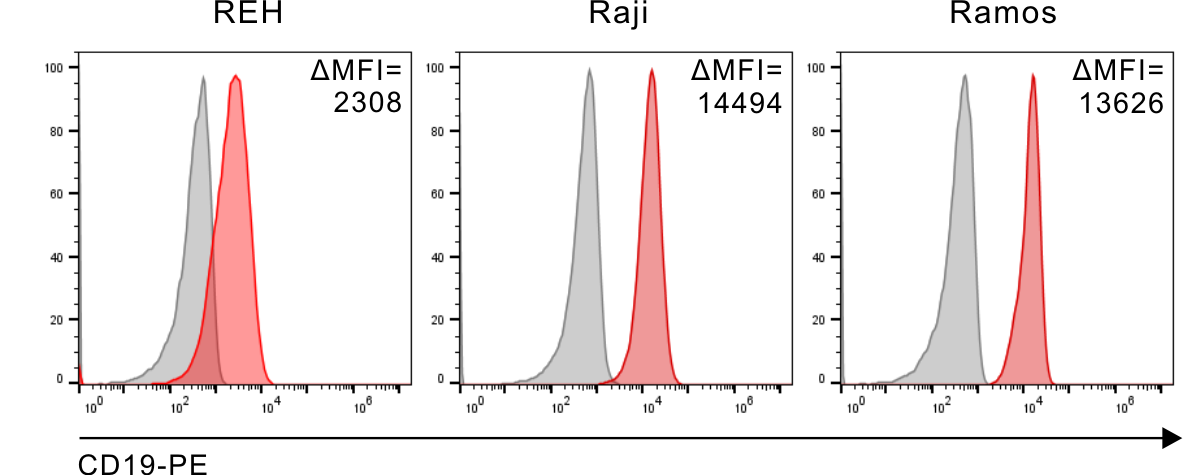


## **Supplemental Figure 2. Assessment of CD19 expression on target cells.**

Surface expression of CD19 on REH, Raji, or Ramos was determined by flow cytometry-based staining with anti-CD19 antibody. Shown is the MFI of CD19 expression on the indicated target cells relative to the MFI of the isotype control (ΔMFI). Data are representative of three independent experiments.


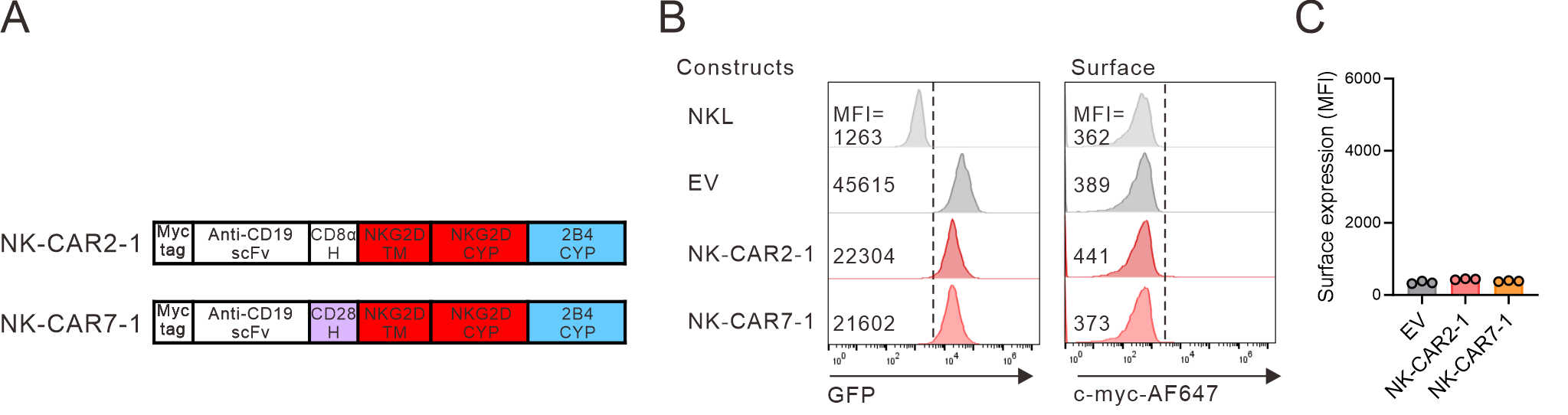


## **Supplemental Figure 3. Effect of NKG2D CYP domain on the expression of NK-CARs.**

(**A**) Schematic diagram of the CAR constructs with the indicated combinations of H and NKG2D domains with 2B4 CYP domain. (**B**) Representative flow cytometry analysis showing the MFI of GFP (left panel) and surface CAR (right panel) expression in CAR NKL cells. (**C**) Summary graph showing the MFI of surface CAR expression in CAR-transduced NKL cells. Data are representative of three independent experiments.


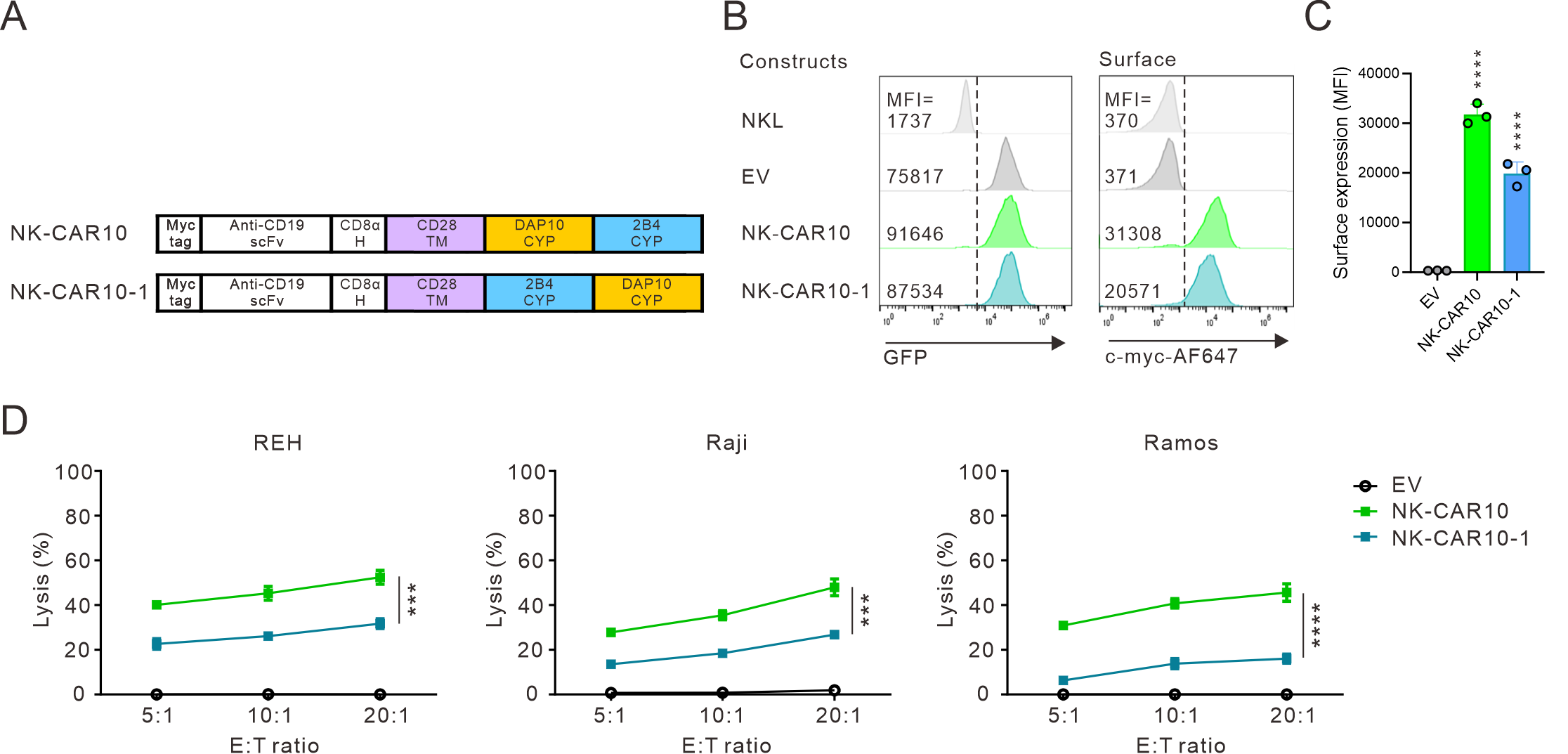


## **Supplemental Figure 4. Effect of sequential order of DAP10 and 2B4 CYP domains on the antitumor activity of NK-CARs.**

(**A**) Schematic diagram of the CAR constructs with the indicated combinations of H and TM domains coupled to different order of DAP10 and 2B4 CYP domain. (**B**) Representative flow cytometry analysis showing the MFI of GFP (left panel) and surface CAR (right panel) expression in CAR NKL cells. (**C**) Summary graph showing the MFI of surface CAR expression in CAR-transduced NKL cells. (**D**) Comparing lysis of REH, Raji, or Ramos cells by the indicated CAR-expressing NKL cells at the indicated E:T ratio. Data were analyzed using the one-way (C) or two-way ANOVA (D) with Dunnett’s multiple comparison test. ****P* < 0.001; *****P* < 0.0001 relative to EV NKL cells (C). Data are representative of three independent experiments.


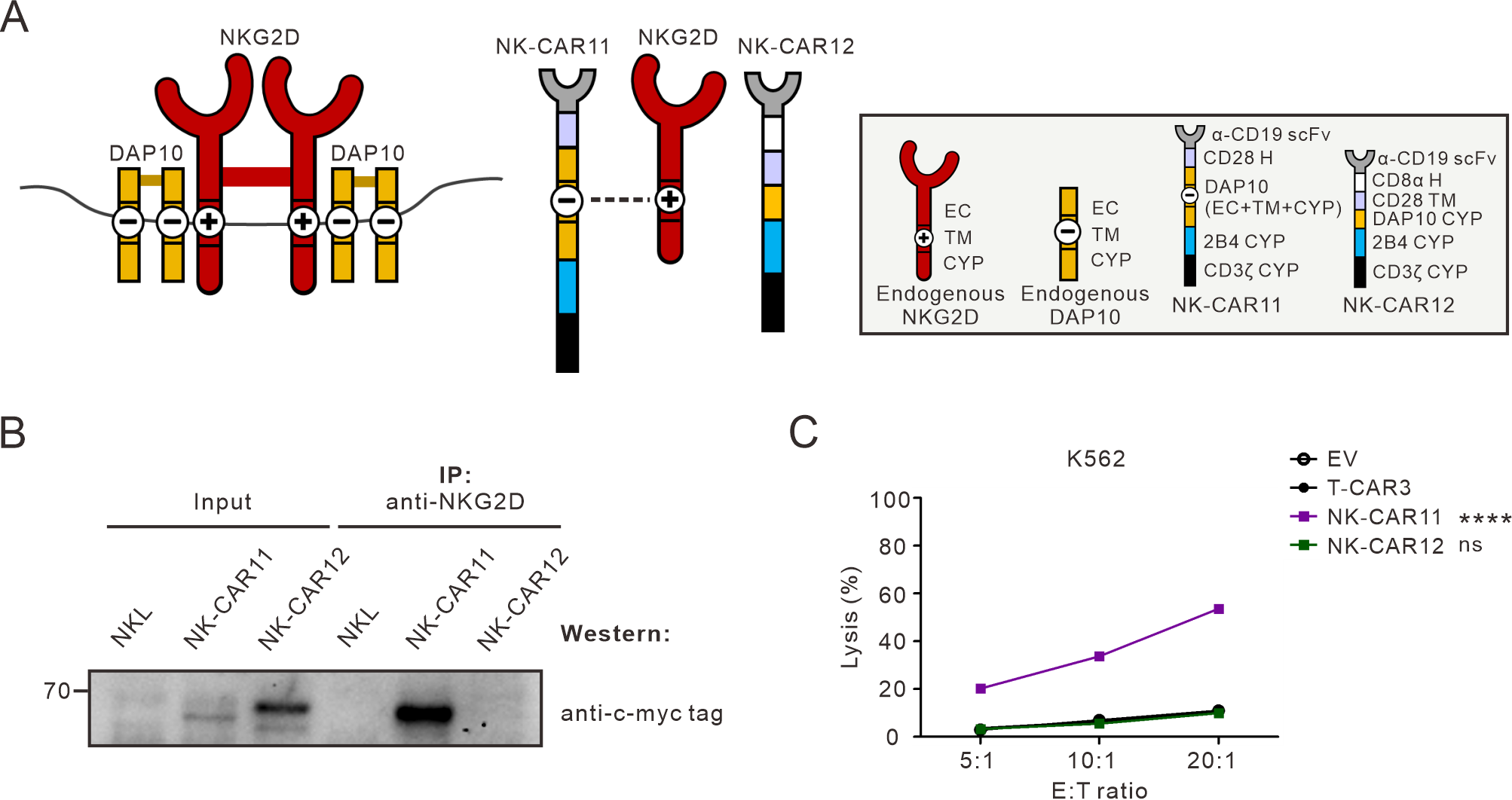


## **Supplemental Figure 5. NK-CAR11 harboring DAP10 TM but not NK-CAR12 associates with endogenous NKG2D receptor.**

(**A**) Scheme for the reported interaction of endogenous NKG2D with endogenous DAP10 through ionic bonds in the TM (left), and the putative interaction of endogenous NKG2D with DAP10 (TM)-containing CAR11 but not CAR12 without DAP10 (TM) (right). (**B**) Endogenous NKG2D interacted with NK-CARs was immunoprecipitated from the lysates of NKL or CAR-NKL cells. Thereafter, immunoblot analysis of the input (5% of the whole cell lysates) and the cell precipitates was performed using anti-c-myc tag antibody. (**C**) NKL and K562 cells were co-cultured at the given E:T ratio for 2 h, and CD19-independent cell killing activity was measured by europium assay. Data were analyzed using the two-way ANOVA with Dunnett’s multiple comparison test (C). ns, not significant; *****P* < 0.0001 relative to T-CAR3 NKL cells. Data are representative of three independent experiments.


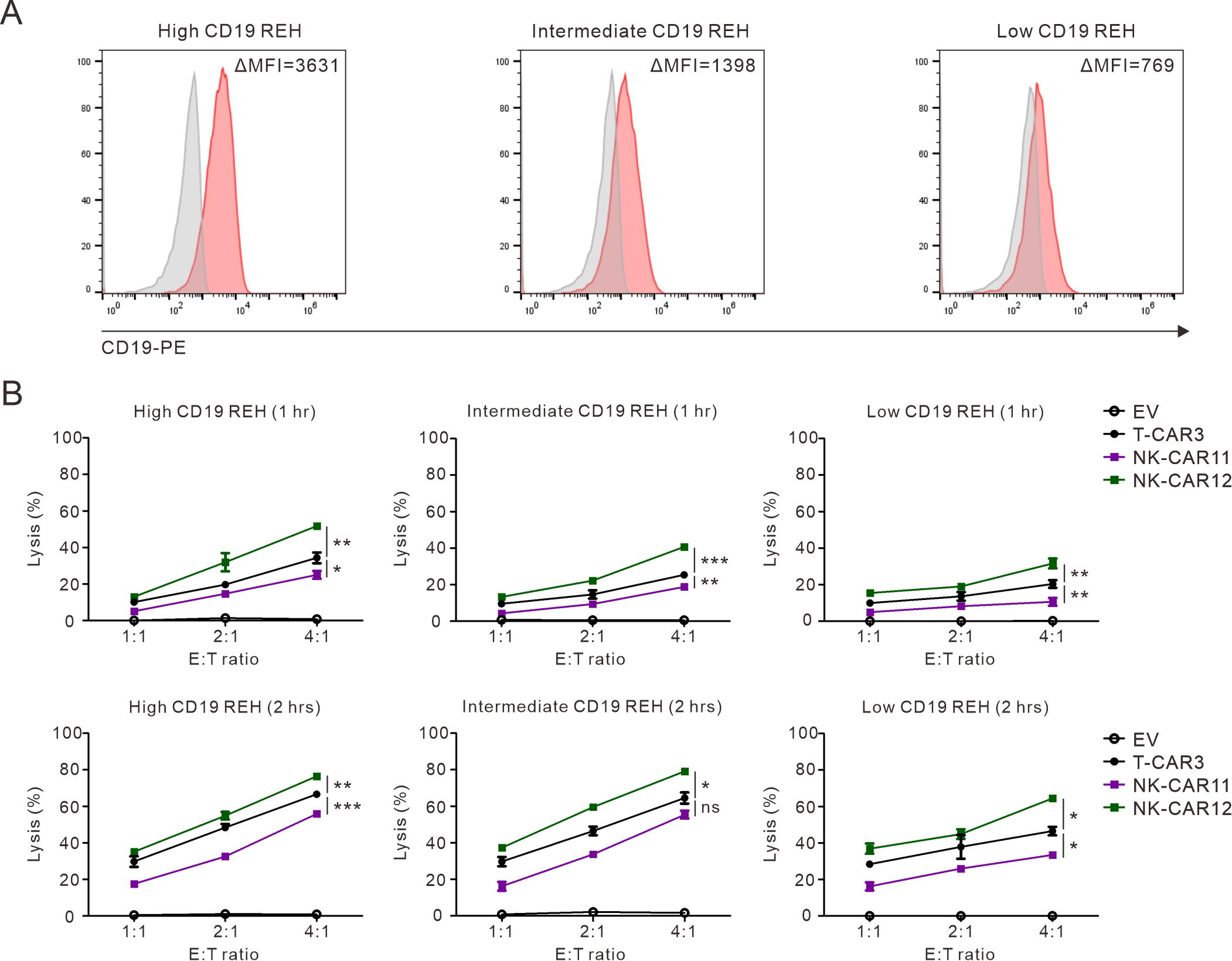


## **Supplemental Figure 6. NK-CAR12 retains superior activity against target cells expressing low levels of CD19.**

(**A**) Validation of REH cells sorted with the different levels of surface CD19 expression by flow cytometry. Shown is the MFI of CD19 expression on the sorted cells relative to the MFI of the isotype control (ΔMFI). (**B**) NKL and the indicated REH cells were co-cultured for 1 h (top) or 2 h (bottom) at the given E:T ratios, and specific cytotoxicity against the different REH cells was measured by europium assay. Data were analyzed using the two-way ANOVA with Dunnett’s multiple comparison test (B). ns, not significant; **P* < 0.05; ***P* < 0.01; ****P* < 0.001 relative to T-CAR3 NKL cells. Data are representative of three independent experiments.


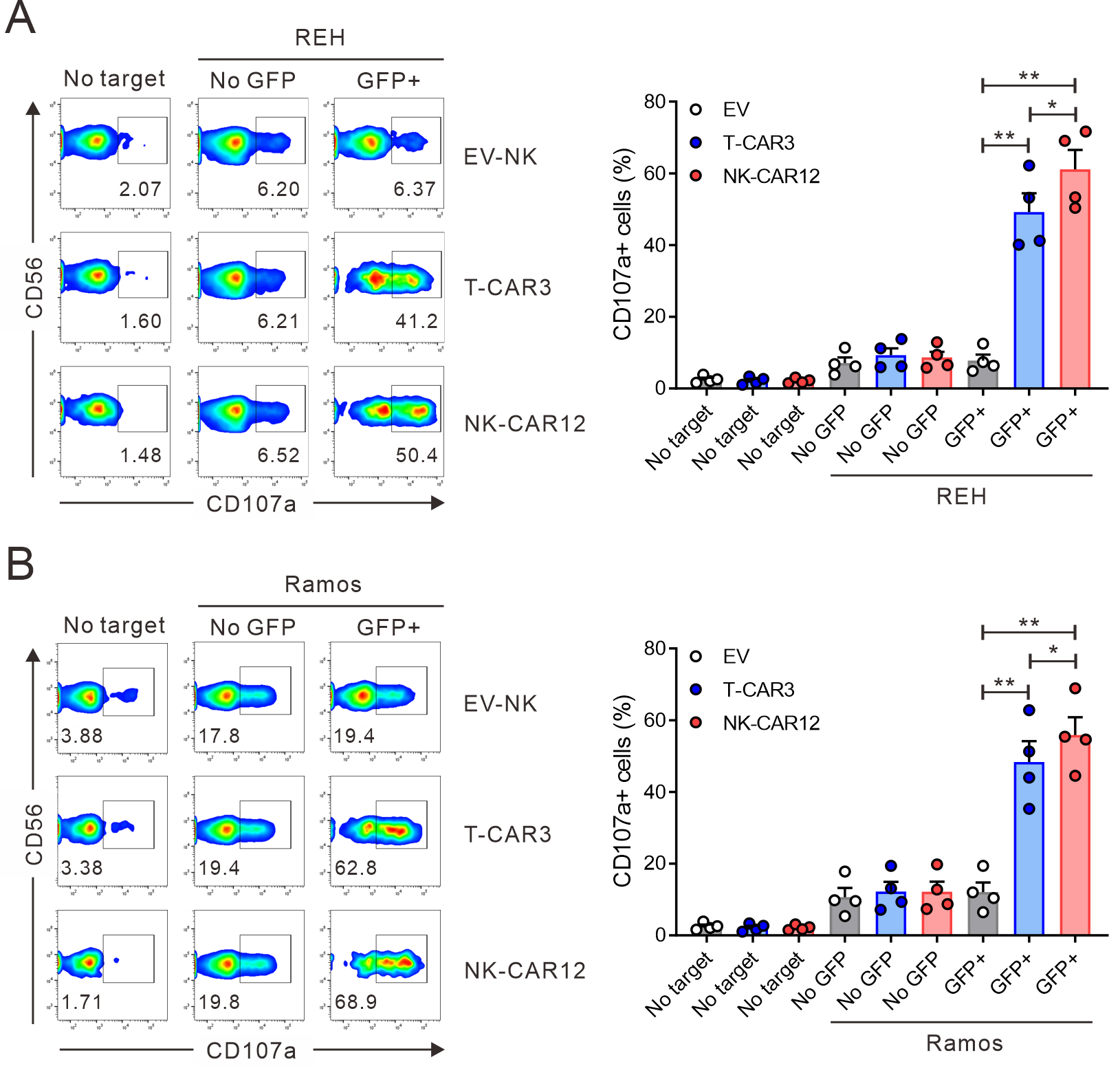


**Supplemental Figure 7. Potent antitumor activity of NK-CAR12 in primary human NK cells.**

(**A** and **B**) Primary human NK cells (*n* = 4 each group) transduced with EV, T-CAR3, or NK-CAR12 construct co-expressing GFP were incubated with REH cells (A) or Ramos cells (B) for 2 h at an E:T ratio of 4:1. Representative FACS profiles (left) and summary graphs (right) showing the percentage of CD107a^+^ primary CAR NK cells in GFP-negative and GFP-positive cells. Values represent the means ± SD; each dot represents an individual donor. Data were analyzed using one-way ANOVA with Tukey’s multiple comparison test. ns, not significant; **P* < 0.05; ***P* < 0.01.


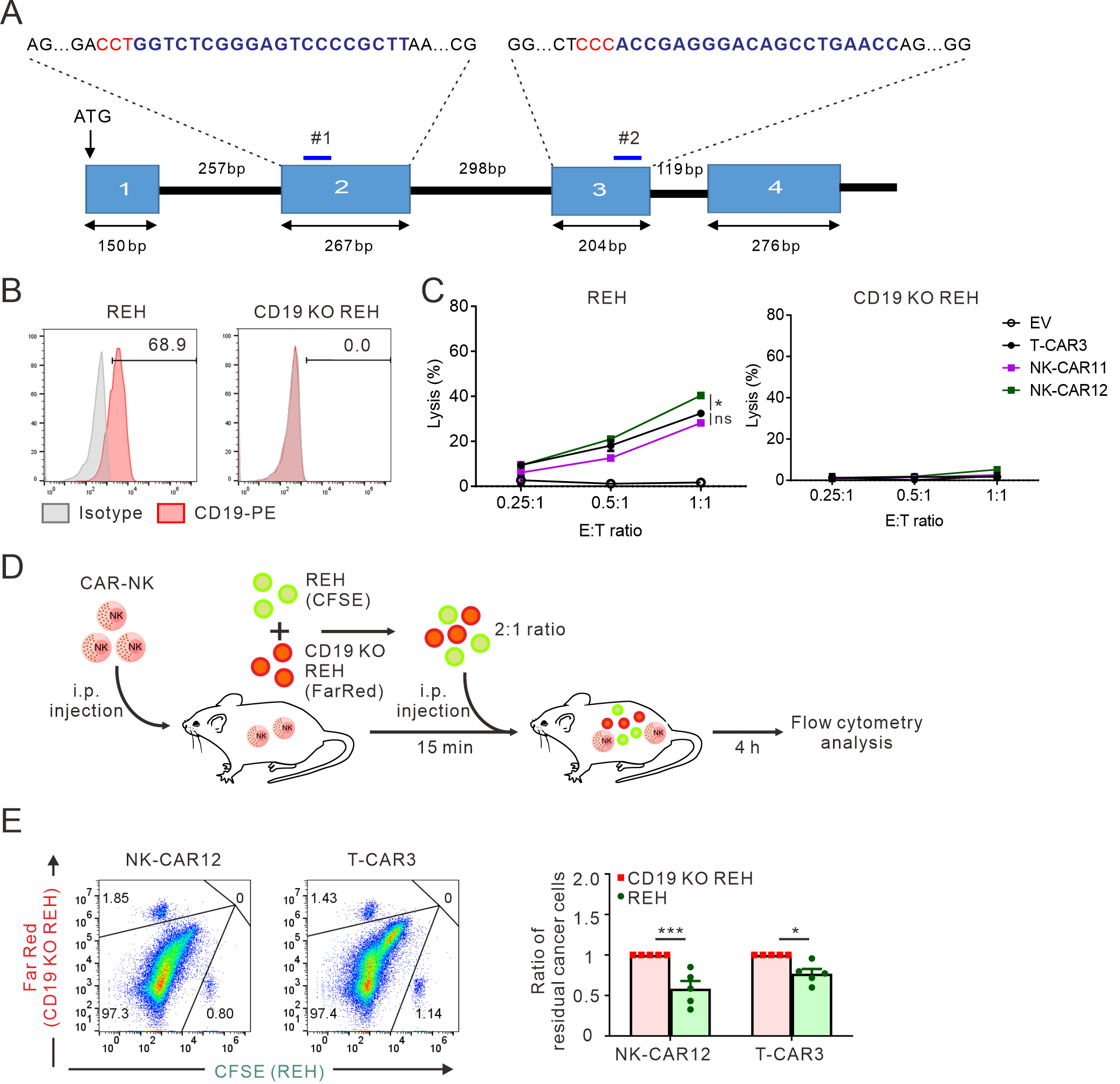


## **Supplemental Figure 8. Generation of CD19 KO REH cells and their use in in vivo leukemia clearance assay.**

(**A**) Scheme of CRISPR/Cas9-mediated knockout of CD19 using specific gRNA #1 or gRNA #2. Two gRNAs with target sequences in exon 2 and exon 3 were selected to delete CD19, respectively. The target sites of the selected gRNAs are indicated in the blue bar, along with the 20-nt target sequences (blue). The PAM sequence is denoted in red. (**B**) Validation of the CD19 KO in REH cells. REH control and CD19 KO REH cells (ΔCD19#1) were harvested, and the surface expression of CD19 was determined by flow cytometry. Shown is the representative FACS profiles with the percentage of the CD19 expression (red shaded histograms). Isotype control staining is shown as gray shaded histograms. (**C**) CAR NK92 and REH cells were co-cultured for 30 min at the given E:T ratios, and specific cytotoxicity against the REH cells was measured by europium assay. (**D**) In vivo leukemia rejection protocol. Immune-deficient NRGA mice received an i.p. injection with CAR NK92 cells. For target cell preparation, REH cells were labeled with CFSE, while CD19 KO REH cells were labeled with FarRed. A 1:1 target cell mix was then injected i.p. into NRG mice 30 min post-injection of NK cells, and the rejection of REH cells relative to CD19 KO REH cells in the peritoneal cavity was measured by flow cytometry after 4 h. (**E**) Flow cytometric analysis of CFSE-labeled REH and FarRed-labeled CD19 KO REH cells recovered from the peritoneal cavity of NRG mice 4 h post-injection to assess NK cell killing activity in vivo. Representative flow cytometry profile (left) and summary graph showing ratio of residual leukemia cells (right). Values represent the means ± SEM; each dot represents an individual mouse. Data were analyzed using the two-way ANOVA with Dunnett’s multiple comparison test (C) or Mann–Whitney *U*-test (E). ns, not significant; **P* < 0.05; ***P* < 0.01; ****P* < 0.001 relative to T-CAR3 NK92 cells (C).
